# Supplementary material for: ‘Where have all the doctors gone?’ A protocol for an ethnographic study of the retention problem in emergency medicine in the UK
Source: BMJ Open. 2020 Nov 30;10(11):e038229. doi: 10.1136/bmjopen-2020-038229 (PMC7705583; doi:10.1136/bmjopen-2020-038229)
Supplement: Author's manuscript [file bmjopen-2020-038229.draft_revisions.pdf]

BMJ Open is committed to open peer review. As part of this commitment we make the peer review history of every article we publish publicly available.

When an article is published we post the peer reviewers' comments and the authors' responses online. We also post the versions of the paper that were used during peer review. These are the versions that the peer review comments apply to.

The versions of the paper that follow are the versions that were submitted during the peer review process. They are not the versions of record or the final published versions. They should not be cited or distributed as the published version of this manuscript.

BMJ Open is an open access journal and the full, final, typeset and author-corrected version of record of the manuscript is available on our site with no access controls, subscription charges or pay-per-view fees (http://bmjopen.bmj.com).

If you have any questions on BMJ Open's open peer review process please email info.bmjopen@bmj.com

BMJ Open

'Where have all the doctors gone?' A protocol for an ethnographic study of the retention problem in Emergency Medicine

Journal:	*BMJ Open*
Manuscript ID	bmjopen-2020-038229
Article Type:	Protocol
Date Submitted by the Author:	03-Mar-2020
Complete List of Authors:	Darbyshire, Daniel; Lancaster University Faculty of Health and Medicine, Medical School; Salford Royal NHS Foundation Trust, Emergency Department<br>Brewster, Liz; Lancaster University Faculty of Health and Medicine, Medical School<br>Isba, Rachel; Lancaster University Faculty of Health and Medicine, Medical School; North Manchester General Hospital, Children's Accident and Emergency Department<br>Body, Richard; The University of Manchester, Division of Cardiovascular Sciences; Manchester University NHS Foundation Trust, Emergency Department<br>Goodwin, Dawn; Lancaster University Faculty of Health and Medicine, Medical School
Keywords:	ACCIDENT & EMERGENCY MEDICINE, Health policy < HEALTH SERVICES ADMINISTRATION & MANAGEMENT, MEDICAL EDUCATION & TRAINING, QUALITATIVE RESEARCH

SCHOLARONE™
Manuscripts

I, the Submitting Author has the right to grant and does grant on behalf of all authors of the Work (as defined in the below author licence), an exclusive licence and/or a non-exclusive licence for contributions from authors who are: i) UK Crown employees; ii) where BMJ has agreed a CC-BY licence shall apply, and/or iii) in accordance with the terms applicable for US Federal Government officers or employees acting as part of their official duties; on a worldwide, perpetual, irrevocable, royalty-free basis to BMJ Publishing Group Ltd ("BMJ") its licensees and where the relevant Journal is co-owned by BMJ to the co-owners of the Journal, to publish the Work in this journal and any other BMJ products and to exploit all rights, as set out in our licence.

The Submitting Author accepts and understands that any supply made under these terms is made by BMJ to the Submitting Author unless you are acting as an employee on behalf of your employer or a postgraduate student of an affiliated institution which is paying any applicable article publishing charge ("APC") for Open Access articles. Where the Submitting Author wishes to make the Work available on an Open Access basis (and intends to pay the relevant APC), the terms of reuse of such Open Access shall be governed by a Creative Commons licence – details of these licences and which Creative Commons licence will apply to this Work are set out in our licence referred to above.

Other than as permitted in any relevant BMJ Author's Self Archiving Policies, I confirm this Work has not been accepted for publication elsewhere, is not being considered for publication elsewhere and does not duplicate material already published. I confirm all authors consent to publication of this Work and authorise the granting of this licence.

For peer review only

Title Page

Title

‘Where have all the doctors gone?’ A protocol for an ethnographic study of the retention problem in Emergency Medicine

Corresponding author: Dr Daniel Darbyshire, B32 Furness College, Lancaster University, Bailrigg, LA1 4YG. d.darbyshire@lancaster.ac.uk (+44)0 7757119141

Authors and affiliations

Daniel Darbyshire (1,2), Liz Brewster (1), Rachel Isba (1,3), Richard Body (4,5), Dawn Goodwin (1)

(1) Lancaster Medical School, Bailrigg, UK. (2) Emergency Department, Salford Royal Hospital NHS Foundation Trust, Salford, UK. (3) Children’s Accident and Emergency Department, North Manchester General Hospital, Manchester, UK. (4) Division of Cardiovascular Sciences, The University of Manchester, Manchester, UK. (5) Emergency Department, Manchester University NHS Foundation Trust, Manchester, UK

Word count = 3978

Keywords - Ethnography, staffing, emergency medicine, sustainability.

Abstract

Introduction

‘Emergency medicine (EM) in the United Kingdom (UK) has a medical staffing crisis.’ Inadequate staffing, in EM and across healthcare, is a problem that affects the quality of patient care globally. Retention of doctors in EM is a particularly acute problem in the UK’s National Health Service (NHS). Sustainable careers in healthcare are gaining increasing attention at a national and international policy level, but research to understand the factors that facilitate retention is lacking.

This study aims to develop understanding of what drives retention of doctors in EM by focusing on those who stay in these careers. By addressing the problem of retention in a different way, using innovative methods in this context, we aim to develop a deeper and more nuanced understanding of sustainable careers in EM.

Methods and analysis

This is an ethnographic study combining participant observation in two emergency departments, interviews with doctors from these departments, from organisations with influence or interest at a policy level and with doctors who have left emergency medicine. The analyses will integrate detailed workplace observation alongside key academic and policy documents using reflexive thematic analysis.

Ethics and dissemination.

Approvals have been obtained from the University Research Ethics Committee and NHS Health Research Authority. The findings will inform understanding of sustainable careers in EM and may be transferable to other settings, professions, and locations that share key characteristics with EM such as paediatrics, emergency nursing, and general practice.

FUNDED BY NIHR National Institute for Health Research

For peer review only

Findings will be disseminated through a series of academic publications and presentations, through local and specialty research engagement, and through targeted policy statements.

Article summary

- Focuses on a national research priority and a global problem.
- Takes a novel approach to understanding the retention problem.
- Engaged with policy makers and the public throughout the research.
- Some aspects of retention will vary by context.
- Policies and practice will evolve during the research, making it harder to unpick some of the findings.

For peer review only

FUNDED BY NIHR | National Institute for Health Research

Main Text

Main text separated under appropriate headings and subheadings using the following hierarchy: BOLD CAPS, bold lower case, Plain text, Italics

INTRODUCTION

The specialty of emergency medicine (EM) provides care across the spectrum of illness and injury to people of all ages. It operates all day, every day and in the United Kingdom (UK) has seen demand increase year on year, with declining performance in terms of patients having their emergency department (ED) visit concluded within four hours, and a rise numbers of patients spending greater than 12 hours in the emergency department.[1] Much of this declining performance is attributed to factors outside of the ED, with exit block due to insufficient hospital beds and community services being the most frequently cited contributing factors.[2,3] Arguably the most important factor for high-quality care in the ED that departments have some control over, is staffing.

Unfortunately, 'Emergency Medicine in the UK has a medical staffing crisis.'[4] Starting with the formation of the Emergency Medicine Taskforce, set up in 2011,[4] the specialty has sought to change practice, policy and culture though innovations such as the 'less than full time training' pilot,[5] increasing recognition of the particular stresses a career in emergency medicine brings and a focus on sustainable careers.[6,7] This has led to emergency medicine growing rapidly in terms of both consultant and trainee numbers but demand for care has outstripped staffing growth and looks to continue to do so.[8]

The EM workforce problem is occurring in the context of staffing shortages in much of the United Kingdom's National Health Service (NHS)[9] and on a background of demand outstripping growth in the global healthcare workforce.[10] The picture is complex. The NHS employs around one million people, with the workforce increasing by around 1.8% from July 2017 to 2018. However, organisations report collective vacancies across the NHS in the region of 100,000 and falling numbers of key staff groups – General Practitioners (GPs) for example – decreased by 1.6% in 2018.[9] The *NHS Long Term Plan*,[11] published in January 2019 (updated August 2019), followed by the *Interim People Plan*,[12] outlined the UK Government's proposed solutions for staffing the NHS. The plans – which focused on staff development, empowerment, and improving leadership culture – were broadly welcomed.[13] There were, however, concerns, for example about the time lag between increasing training numbers and generating qualified practitioners in employment, with potential over-reliance on international recruitment in the interim.[13,14]

From recruitment to retention

EM historically had a recruitment problem, but after much work by the then College of Emergency Medicine (now the Royal College of Emergency Medicine) and Health Education England, amongst others, in improving the image of the specialty and the experience of trainees, fill rates for EM emergency medicine training posts reached 96% in 2011.[15] Recruitment has since fallen from the 2011 high, yet it remains strong and competitive, when compared with other specialities, at 86% in 2019 (compared to 77% for internal medicine and 69% for paediatrics).[16] However, attrition from training programmes is unsustainably high, with The King's Fund (an independent English think tank) reporting that:

For peer review only

FUNDED BY NIHR National Institute for Health Research

‘problems with retention mean it [emergency medicine] has the greatest attrition rate of any medical specialty, with almost 50 per cent of registrar doctors in their third or fourth year of training resigning.’[17]

Following on from a 2014 survey of trainees that looked at the attractiveness of a career in EM,[18] the Emergency Medicine Trainees’ Association now conduct its own annual surveys.[19–21] These surveys elucidate the challenges faced by trainees, specifically burnout, poor working conditions, and workload pressures. This is supported by the General Medical Council’s national training survey which showed EM as the specialty with the highest work intensity[22] and by the high attrition from the emergency medicine specialty training programme.[23] The data on trainees is reflected for consultants and staff and associate specialist grades, with all groups being undersubscribed and experiencing exodus.[23]

Recruiting and retaining EM clinicians is fundamentally connected to the provision of high-quality clinical care. More experienced and senior doctors deliver better care, performing fewer unnecessary tests and getting fewer complaints, and experience is only gained by retaining doctors within the specialty. Retention of doctors affects wider health and care services in two main ways. Firstly, the presence of doctors with more experience results in efficiency savings both in the ED and downstream in a patient’s hospital journey, with recent studies finding a greater than 20% reduction in admission rates, reduced waiting times, and cost savings of £3 million to one hospital.[24,25] The introduction of the Major Trauma Networks in 2010 with consultant-led care of severely injured patients in London saved an estimated 58 lives in the first year.[26] Secondly, staff turnover is costly and disruptive. When ED staff leave it is highly unusual for replacements to be found quickly, if at all, often leading to locum (temporary) doctors filling in who are ‘generally less efficient, and less safe, than permanent members of staff’.[27] Costs in the UK are not clear but an approximate figure for replacing one fully trained emergency physician in the US is $164,000.[28]

We would argue that the evidence is therefore clear that staff retention is important for quality emergency care. What is not clear is what drives retention and what allows people to have sustainable careers. This knowledge gap was also identified in the 2017 James Lind Alliance (see figure 1) priority setting partners

For peer review only

FUNDED BY NIHR | National Institute for Health Research

The James Lind Alliance

The James Lind Alliance (JLA) is a non-profit making initiative established in 2004. It brings patients, carers and clinicians together in Priority Setting Partnerships (PSPs) to identify and prioritise the Top 10 unanswered questions or evidence uncertainties that they agree are the most important.

The aim of this is to make sure that health research funders are aware of the issues that matter most to the people who need to use the research in their everyday lives. It is part of the NIHR (National Institute for Health Research), the research funding arm of the United Kingdom's NHS (National Health Service).

Figure 1. What is the James Lind Alliance? Information taken from the JLA website. http://www.jla.nihr.ac.uk/about-the-james-lind-alliance/ (accessed 15th January 2020)

hip for EM research. Number four in their list of the top ten research priorities in EM asked: 'with regard to how ED staff development is managed, what initiatives can improve staff engagement, resilience, retention, satisfaction, individuality and responsibility?'[29] This priority was supported by calls from various other stakeholders to address the issue of retention including The King's Fund,[17,30] Health Education England[15], and the Royal College of Emergency Medicine.[27, 31–33]

The proposed study aims to address retention – an innovative approach, contrasting with previous work that has focused predominantly on why people leave. The ethnographic methodology is also innovative in this setting as the retention problem has not previously been explored using this approach. By developing fresh insights into the complex and interacting factors that positively influence retention of doctors in emergency medicine, we hope to inform future efforts aimed at reducing exodus from the specialty, at both a research and policy level.

Research aim and objectives

The aim of this study is to gain a deep understanding of retention in emergency medicine, in order to elucidate how retention is made possible. This aim requires a study design that can tackle complexity and develop new understandings. The objectives are to:

- understand in detail the day-to-day lived experience of EM doctors, to identify and explore factors influencing retention;
- situate these descriptions within the current educational and health policy contexts;
- advance the debate and make policy and practice recommendations based on a detailed understanding of retention of doctors in EM.

FUNDED BY NIHR | National Institute for Health Research

METHODS

Overview

We will conduct an ethnographic study of emergency medicine doctors in two type 1 emergency departments in the North West of England. Ethnography is derived from anthropology and social sciences and is historically associated with gaining cultural understandings about groups, with the origins of the practice going back to colonial-era studies of 'exotic' civilisations.[34] Its use within health research is broad and well-established, from a 1961 study of medical student culture[35] through to a recent study trying to understand why one particular maternity unit performs highly.[36]

Ethnography is 'a systematic approach to learning about social and cultural life of communities, institutions, and other settings'.[37] Key features of ethnography relevant to this study are that it is investigative, the primary tool of data collection is the researcher, and that it 'emphasises and builds on the perspective of the people in the research setting'.[37]

Ethnography creates understanding through description and analysis. This usually involves going to a place and collecting data – which is often a detailed description of what happens in that place along with the accompanying talk – over a period of time. What information is recorded, how long the researcher spends there, and what other forms of data are collected, depends on the context of the research and question being asked. For this research, ethnography refers to the totality of the study including the time spent observing in the department (referred to as participant-observation hereafter), interviews, and policy analysis.

This ethnographic study will involve the lead researcher spending time in two different EDs, trying to understand what allows doctors to have sustainable careers. This will involve observing the people, space, and happenings in the departments, and speaking to those in these spaces, keeping the focus on retention. This will be supported by interviews with doctors (conducted away from the department) and with others who can inform the research question, and by critical review of policy and academic literature relating to retention. The literature search strategy, in the form of a scoping review, is described elsewhere.[38] Each step in the process is expanded on below and summarised in figure 2.

FUNDED BY NIHR National Institute for Health Research

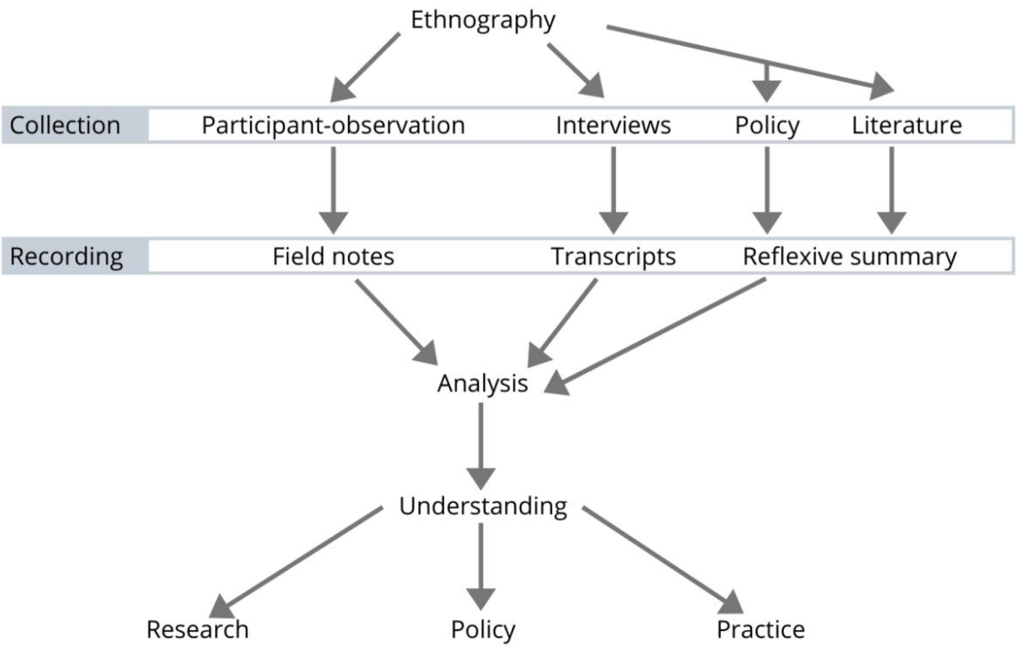

Figure 2. Summary of the ethnography

This study subscribes to the 'ethnographic paradigm'[39], by this we mean that we don't see value in predetermining a particular way of seeing the world, and agree with Atkinson when he writes that 'ideas can and should come from a variety of sources'.[40] This study draws on the sociological practice of ethnography and as such the findings will be analysed, interpreted, and challenged through a number of sociological theories. These will include, but are not limited to, theories of work[41,42] such emotional labour[43] and social capital,[44] theories of community such as Tönnies discussion of Gemeinschaft and Gesellschaft (community and society)[45], and Bourdieu's conception of symbolic power.[46] This list is not intended to be prescriptive – it aims to show theories that may prove informative.

As the study is built on the interpretation of DD (the researcher collecting the data and performing the analysis), a discussion of their positionality will be included when the study reports.[47] This is an integral part of reflexive thematic analysis described below, 'addressing threats to credibility in ethnography requires different techniques from those used in experimental studies.'[48] Our study aims to produce a clear audit trail, of which publishing this protocol is a part.[38,49] Other important parts of the audit trail are notes made during observations about why the researcher has chosen to observe at particular times and locations, documents generated around meetings of the research team, and documents from peer-review, ethics, and the funder which have shaped the study.[49] We are aiming for transferability not generalisability, reflecting the importance of context,[50] and will utilise reflexive thematic analysis, sense checking from the public involvement group. We aim for confirmability by aligning interpretations and data in the final report.[51]

Patient and public involvement

For peer review only

The public has been involved in the development of this study at several levels and at different key points in the development of the protocol. The aforementioned James Lind Alliance priority setting exercise demonstrated the public's belief that the topic areas is important.[29] To confirm this, with the support of the NIHR Research Design Service North West and the Research and Development Department at Blackpool Victoria Hospital, we ran a public involvement event. During this event, members of the public agreed with the James Lind Alliance that exploring factors related to the retention of doctors in EM merited investigation. Participants thought the proposed methods were acceptable to patients, but questioned whether ethnography would be seen as acceptable to a clinical audience. A member of the initial patient involvement group has volunteered to be further involved in the study by becoming a member of the steering committee and sense-checking the conclusions we gather during the scoping review part of the study.[38]

As the main focus of the study is EM doctors, we also sought their views as part of our engagement strategy. We presented the research plan, along with some of the preliminary findings from the scoping review,[38] at the Emergency Medicine Trainees' Association National Conference in Cardiff, in November 2018. Feedback was obtained during the question and answer question and by providing a link to an online form. This process was repeated at the Oxford Society for Emergency Medicine Conference and the Association for the Study of Medical Education Researching Medical Education Meeting in London, both in December 2018. Thirteen attendees provided written feedback, including one offer to help, which led to recruitment of a second reviewer for the scoping review, with many more providing verbal comments and questions.

Participant-observation

Participant-observation refers to the time spent in the research setting watching, listening, and documenting. It is strongly linked with ethnography and is sometimes synonymous with field work. These terms are contentious[52] and for clarity we refer to participant-observation as the component of ethnography that occurs in the research setting. All data collection will be conducted by DD who will undertake observation at two sites, each for a period of twelve weeks. At each site they will perform observations in all parts of the department and at all times of the day. A day of participant-observation will be divided in two. The first half of the day will be spent conducting observations and writing field notes, incorporate talking with staff and documenting what they say, and the researcher's thoughts about the encounter. We will investigate the working environment, interaction between staff, and behaviours related to retention. The field notes will be handwritten in a notebook. The other half of the day will involve typing up, reflecting on, and expanding field notes. We aim to perform four days of observation each week by scheduling forty-eight days over the twelve-week period with start times reflecting the shift patterns locally, but to include days, evening, nights and weekends. A total of twenty-four weeks' participant-observation was chosen as it will allow DD to participate in observations at all times, with flexibility to repeat observations at key times or with specific key informants. Traditionally ethnographic observation has been conducted over a much greater period of time than the one that proposed, to allow for a period of familiarisation. This process will be accelerated in this study due to DD's pre-existing knowledge of EM and EDs. This more focused form of ethnography has a developing literature for its use in complex healthcare settings[53] and

For peer review only

For peer review only - http://bmjopen.bmj.com/site/about/guidelines.xhtml

based on this work, and the intensity of the observation within the twelve-week period, it is estimated that this will be sufficient to meet the aims of the study.

Interviews

In addition to the participant-observation conversations described above, we will conduct interviews, reserving the term for the physically separate, planned, audio-recorded and transcribed encounters. DD will conduct all the interviews, aiming to complete around 40. Ten interviews with doctors across all levels of seniority from each of the two research sites, ten interviews with individuals within key organisations relevant to retention in EM (such as the Royal College of Emergency Medicine, the Emergency Medicine Trainees' Association, Health Education England, and NHS Improvement), and ten with doctors who have left EM. Potential participants for interviews will be identified during the periods of participant-observation and the literature review and supplemented by recommendations obtained from interviewees. Interviewing ten doctors from each research site would represent between a quarter and a third of doctors at each level present on a rota at any time. The sample size has been agreed upon by discussion within the research team, who have extensive experience with this methodology, and by consulting key texts, and represents an estimation for when theoretical saturation (see glossary) might be achieved.[54–56]

Interviews will take place in a neutral space which allows for private conversation. The interview guide is available in Appendix 1. However, it is expected to evolve as the study progresses, based on pilot interviews, and ongoing analysis. Interviews will be digitally recorded, transcribed, and imported into nVivo 12,[57] the qualitative data analysis computer package that facilitates the organisation and analysis of research data. After each interview DD will record reflections in the field notes.

Policy and literature

Policy documents (grey literature) will be identified through the aforementioned scoping review[38] and from the other aspects of the ethnography. Key documents will be chosen based on relevance to research context and incorporated into the analysis. A similar process will be used for academic literature.

Inclusion criteria

Participant observation sites were selected based on the following factors:

- presence of foundation, core, and higher trainees;
- presence of staff and associate specialists;
- Type 1 ED (major ED, providing a consultant-led 24-hour service with full resuscitation facilities).[58]

Interview and observation candidates

Within the two EDs the focus for participant observation, and interviews candidates is:

- doctor;
- working in the study site;
- staff member for at least 4 weeks
- aim to include a maximally inclusive representation of the department, including age, gender, seniority, international medical graduates, and UK trained doctors.

For peer review only

The inclusion criteria are intended to be broad and inclusive. The 4-week criteria were chosen to avoid interviewing very new staff members at the start of their work. The longitudinal nature of the ethnography means that these candidates may be eligible once they have been in the department for a month or more.

Consent

Participant-observation

Informed consent needs to match the research and the environment in which it is being undertaken. We are not alone in making this point, several previous studies within the NHS[36,59] and the ED[60] have taken a proportionate route, clearly demonstrating the acceptability and utility of such an approach.

To ensure participants are informed of the study protocol, aims, and data collection methods, a continuous information sharing approach will be taken, mirroring the studies referenced above. The research has already been explained to the senior leadership at each study site, along with the research and development leads. To complement this, we will present the research plan to the medical and nursing staff at scheduled meetings such as junior doctors teaching, band 5, 6 and 7 nursing meetings, consultant meetings and departmental research and governance meetings. We will advertise the start of the study with posters giving people the opportunity to ask questions, and state objections, before it starts. Emails will be circulated to all ED staff informing them of the study. In addition, we will offer a brief explanation to staff members who the researcher interacts with in the department, as both part of the consent process and as a way to start conversations as part of the research method.

When introducing himself in the research setting DD will reiterate the voluntary nature of the study and that it is ok for them to ask for observation to stop, either temporarily or until further notice. Based on previous studies in the ED this is unlikely to occur frequently, and as such we intend, if this happens, to relocate to a different part of the department to continue observation. Should an individual ask for more information about the study we will provide them with a short summary of the study, a link to this protocol and contact details for the research team.

Capacity (to make decisions for themselves)[61] for staff members is highly unlikely to be an issue. As an EM specialty trainee (registrar, resident), DD is trained and experienced is assessing capacity in a range of settings and scenarios.

Patients are not participants in this study, and no patient identifiable data will be recorded, but they, and other staff members, do occupy the research space. Their confidentiality and acceptance of the research is vital. While we believe it is unfeasible, overly burdensome, and unnecessary to obtain consent from patients transiting through the emergency department, we will not ignore them either. Either DD will introduce themselves or will be introduced by the member of staff they are accompanying. If a patient does not want to have the care they receive observed, DD will withdraw from that episode of care. DD will rely on his experience and expertise of working within this environment to proportionally address this issue in real time.

FUNDED BY NIHR National Institute for Health Research

For peer review only - http://bmjopen.bmj.com/site/about/guidelines.xhtml

Informed consent for ethnographic study is different to other types of research, particularly so in unpredictable settings such as the ED. The use of the standard written consent procedure is overly burdensome to the researcher and participant, not feasible, and would bar patients and staff members who occupy these spaces from benefiting from this type of research.[62]

Interviews

Formal written consent, supported by a participant information sheet, will be obtained for the formal, recorded, interviews. Consent will be obtained by DD prior to conducting the interview. Having expressed initial interest in participating, potential interviewees will be provided with a copy of the consent form and participant information sheet. On the day of the interview, the interviewee will be given another opportunity to review the participant information sheet and consent form, with DD on hand to answer any questions. Some interviewees, especially those with high-profile roles in national organisations, may feel that anonymisation is either impossible or not in their interests; they will be given the option of participating in the study in a non-anonymised fashion. They will retain the right to have their interview anonymised, and all interviewees have the right to withdraw, up to the point of publication.

ANALYSIS

During the data collection phase of the study, initial analysis will take the form of reflective analytical notes recorded in the fieldnotes, these observations and interpretations guide the ongoing analysis. Once data collection is complete, a more thorough, systematic analysis will be completed utilising reflexive thematic analysis.[63–65] This process leads to generation of themes – these are not groups of data that are about the same thing, but 'stories about particular patterns of shared meaning across the dataset'.[63] Reflexive thematic analysis emphasises the effortful nature of analysis, in that themes are not found within the data, rather they are created though analytical work. This highlights the role of the researcher in generating the analysis, laying this open to scrutiny. The analysis will use the six-stage approach to thematic analysis (see Figure 3 for a summary).[63,66] These stages are descriptive and supportive, as opposed to having clear divisions. It is expected that the analysis will go back and forth through the stages multiple times.

Stage	Description	Applied to this study
1	**Familiarisation** – involves becoming immersed in the data, getting to know it. It is about noticing things that may (or may not) be important.	This will involve reading field notes, transcripts, and other documents, and making notes about connections, possibilities, and quirks.
2	**Generating codes** – attaching labels known as codes to portions of data to create meaning at the intersection between the data set and interpretative resource that is the researcher.	Using qualitative data analysis computer software (NVivo 12 for Mac) to attach codes to data. Coding choices will be documented and attached to each code.

FUNDED BY NIHR National Institute for Health Research

For peer review only

3	**Generating initial themes** – a good theme both identifies the area of data and tells the researcher something about it. These themes will evolve or be discarded. Sometimes called candidate themes.	Stepping back form the fine detail of the data to try and group codes that inform a specific issue.
4	**Revising/reviewing themes** – involves reviewing themes against the coded data and the entire data set. Mismatches lead to revisions.	Compiling all data for each candidate theme and reviewing against the coded data and data set in turn.
5	**Defining and naming themes** – creatively attaching names to themes that signal the core of theme. This aims to move analysis to the conceptual level while maintaining links to the data.	Thinking about the audience and research objectively to name themes that can help effectively communicate the findings.
**6**	**Writing** – producing the output from the research. The writing tests the themes. How well do they work together and in isolation? How do they stand up to the literature? These challenges lead to returning to earlier analytical stages.	Writing up the doctoral thesis, papers for medical and sociological audiences, and producing summaries for policy makers and the public.

Figure 3. The six stages of thematic analysis as applied to this study. An iterative process, it is expected that the analysis will go back and forth between stages a number of times.[63,66]

Limitations

The study is geographically limited to the North West of England. Much of the activity of EDs is similar nationally, but local differences will mean the findings will require interpretation and translation to other locales.

This study is focused solely on doctors working in EDs, there may be some transferability to other professions or working environments, but this is a limitation.

Data collection by someone without experience of the ED may lead to different conclusions, though this might be at the expense of access problems and detailed understanding of the work in the ED.[52]

ETHICS AND DISSEMINATION

Approval from Lancaster University via the Faculty of Health and Medicine Research Ethics Committee (FHMREC18058) was received on 15th April 2019. Health Research Authority approval was formalised on 11th November 2019, IRAS number 256306. Traditional academic dissemination will occur locally through seminars and lectures, and to a broader audience though conference presentations and academic publications. Dissemination to the public will be facilitated by presentations at knowledge translation events and by engaging with local media and research translation services such as The Conversation. Policy briefs

FUNDED BY NIHR National Institute for Health Research

For peer review only - http://bmjopen.bmj.com/site/about/guidelines.xhtml

summarising the findings will be provided for key audiences such as NHS England, Health Education England, and the Royal College of Emergency Medicine.

Glossary

Audit trail – a thorough collection of documentation regarding all aspects of the research. Qualitative research often evolves though the iterative process of data collection and analysis, requiring the researcher to make frequent decisions which alter the course of the research. Documents that form this trail of evidence include field notes, a research diary, and correspondence.[49,67]

Ethnography – a method based on description and the key assumption that 'personal engagement with the subject is the key to understanding a particular culture or social setting'.[68] It can involve many different ways of collecting data, but is strongly associated with participant-observation and field work.

Participant-observation – a combination of a wide variety of methods including observation, informal interviews and conversations, combined with analysis of other materials encountered in the research setting.[69]

Positionality – how the researcher is positioned with respect to the social and political context of the study. Affects all aspects from formation of the research question to analysis. Making this explicit allows the reader to interpret the findings in context.[47,70]

Reflexivity – conscious examination and explanation of how the researcher's positionality influences and is influenced by the research as it progresses.[71]

Theoretical saturation – the continuation of sampling and data collection until no new conceptual insights are generated. At this point the researcher has provided repeated evidence for his or her conceptual categories.[72]

Trustworthiness – the ways in which a qualitative researcher ensures quality in their research, specifically in terms of transferability and credibility.[51] Moves discussions about research quality away from the quantitative paradigm of triangulation and generalisability.

Competing interests statement

This publication presents independent research funded by the National Institute for Health Research (NIHR). The views expressed are those of the author(s) and not necessarily those of the NHS, the NIHR or the Department of Health and Social Care.

All authors have completed the ICMJE uniform disclosure form at www.icmje.org/coi_disclosure.pdf and declare: DD had financial support from the NIHR, BMA Foundation and the Royal College of Emergency Medicine for the submitted work; no financial relationships with any organisations that might have an interest in the submitted work in the previous three years; no other relationships or activities that could appear to have influenced the submitted work.

Funding statement

FUNDED BY NIHR National Institute for Health Research

For peer review only

For peer review only - http://bmjopen.bmj.com/site/about/guidelines.xhtml

Daniel Darbyshire is funded by a National Institute for Health Research (NIHR) Doctoral Fellowship for this research project. Initiation of the study was supported by the BMA Foundation Kathleen Harper Award and the RCEM Young Investigator Award.

Author statement

All authors have had substantial contribution to the design and development of this protocol, all have been involved in the drafting and critically revising of the protocol document, all have given final approval of the version to be published, and all agree to be accountable for all aspects of the work in ensuring that questions related to the accuracy or integrity of any part of the work are appropriately investigated and resolved.

Acknowledgements

The authors would like to acknowledge the ongoing work of Gavin Quick and Anne Clark for their public and patient involvement role in developing the protocol.

References

1 Royal College of Emergency Medicine. RCEM Winter flow project: final report July 2019. London, UK: Royal College of Emergency Medicine 2019. https://rcem.ac.uk/docs/RCEM_Winter_Flow_Project_Final_Report_2019.pdf (accessed 11 Oct 2019).

2 Mason S, Knowles E, Boyle A. Exit block in emergency departments: a rapid evidence review. *Emerg Med J* 2017;**34**:46–51. doi:10.1136/emermed-2015-205201

3 Knowles E, Mason SM, Smith C. Factors associated with exit block and impact on the emergency department. *Emerg Med J* 2017;**34**:61–2. doi:10.1136/emermed-2015-205202

4 Hughes G. The emergency medicine taskforce: an interim report. *Emerg Med J* 2013;**30**:348–348. doi:10.1136/emermed-2012-202260

5 Clancy M. The Less Than Full Time (LTFT(3)) working in emergency medicine pilot final report. London, UK: The Royal College of Emergency Medicine 2019. https://www.rcem.ac.uk/docs/Training/LTFT_pilot_FINAL_REPORT-for_website.pdf (accessed 11 Oct 2019).

6 Hewitt S, Kennedy U. EM-POWER: a wellness compendium for EM. London, UK: The Royal College of Emergency Medicine 2019. https://www.rcem.ac.uk/docs/Sustainable%20Working/0.%20Wellness%20Compendium%20(Apr%202019).pdf (accessed 11 Oct 2019).

7 RCEM Sustainable Working Practices Committee. EMPOWER: A guide to engage and retain your established EM staff. London, UK: Royal College of Emergency Medicine 2018. https://www.rcem.ac.uk/docs/Workforce/Engage%20and%20Retain%20Sept%202018.pdf (accessed 20 Aug 2019).

FUNDED BY NIHR National Institute for Health Research

For peer review only

8 Smith E, RCEM Service Design and Configuration Committee. RCEM workforce recommendations 2018: consultant staffing in emergency departments in the UK. The Royal College of Emergency Medicine 2018. https://www.rcem.ac.uk/docs/Workforce/RCEM%20Workforce%20Recommendations%202018%20-%20Consultant%20Staffing.pdf (accessed 6 Feb 2019).

9 Buchan J, Charlesworth A, Gershlick B, *et al.* A critical moment: NHS staffing trends, retention and attrition. London, UK: The Health Foundation 2019. https://www.health.org.uk/sites/default/files/upload/publications/2019/A%20Critical%20Moment_1.pdf (accessed 11 Oct 2019).

10 Britnell M. Human: Solving the global workforce crisis in health care. The Nuffield Trust. 2019.https://www.nuffieldtrust.org.uk/news-item/human-solving-the-global-workforce-crisis-in-health-care (accessed 10 Jun 2019).

11 NHS England. The NHS long term plan. UK: NHS 2019. https://www.longtermplan.nhs.uk/wp-content/uploads/2019/08/nhs-long-term-plan-version-1.2.pdf (accessed 11 Oct 2019).

12 NHS Improvement. Interim NHS people plan. London, UK: NHS Improvement 2019. https://improvement.nhs.uk/resources/interim-nhs-people-plan/ (accessed 10 Jun 2019).

13 Bailey S. The King's Fund responds to the interim NHS people plan. The King's Fund. 2019.https://www.kingsfund.org.uk/press/press-releases/interim-nhs-people-plan (accessed 11 Oct 2019).

14 Campbell J, Dussault G, Buchan J, *et al.* A universal truth: no health without a workforce. Geneva: Global Health Workforce Alliance and World Health Organization 2013. https://www.who.int/workforcealliance/knowledge/resources/GHWA-a_universal_truth_report.pdf (accessed 10 Jun 2019).

15 Health Education England. Emergency medicine: background to HEE proposals to address workforce shortages. Health Education England 2013. https://www.hee.nhs.uk/our-work/emergency-medicine (accessed 6 Feb 2019).

16 Health Education England. Specialty recruitment: round 1 - acceptance and fill rate. Health Education England. 2018.https://www.hee.nhs.uk/our-work/medical-recruitment/specialty-recruitment-round-1-acceptance-fill-rate (accessed 5 Feb 2019).

17 The King's Fund. What's going on in A&E? The King's Fund 2014. https://www.kingsfund.org.uk/projects/urgent-emergency-care/urgentand- emergency-care-mythbusters (accessed 5 Feb 2019).

18 Joy T, Moran J, Hassan T, *et al.* The attractiveness of a career in Emergency Medicine: A survey of UK trainees. The College of Emergency Medicine 2014. http://www.emtraineesassociation.co.uk/emta-surveys.html# (accessed 5 Feb 2019)

For peer review only

FUNDED BY NIHR National Institute for Health Research

19 Archer K, Bailey J, Jenkinson E. EMTA trainee survey 2015. Emergency Medicine Trainees Association 2016. http://www.emtraineesassociation.co.uk/emta-surveys.html# (accessed 4 Feb 2019).

20 Bailey J, Archer K, Stewart P, *et al.* EMTA survey 2016. Emergency Medicine Trainees Association 2017. http://www.emtraineesassociation.co.uk/emta-surveys.html# (accessed 4 Feb 2019).

21 Bailey J, Mashru A, Stewart P, *et al.* EMTA survey 2017. Emergency Medicine Trainees Association 2018. http://www.emtraineesassociation.co.uk/emta-surveys.html# (accessed 4 Feb 2019).

22 General Medical Council. National training survey 2019: Initial findings report. Manchester, UK: GMC 2019. https://www.gmc-uk.org/-/media/national-training-surveys-initial-findings-report-20190705_2.pdf?la=en (accessed 11 Oct 2019).

23 Health Education England, NHS England, NHS Improvement, *et al.* Securing the future workforce for emergency department in England. NHS Improvement 2017. https://improvement.nhs.uk/documents/1826/Emergency_department_workforce_plan_-_111017_Final.3.pdf (accessed 28 May 2019).

24 White AL, Armstrong PAR, Thakore S. Impact of senior clinical review on patient disposition from the emergency department. *Emergency Medicine Journal* 2010;**27**:262–5. doi:10.1136/emj.2009.077842

25 Geelhoed GC, Geelhoed EA. Positive impact of increased number of emergency consultants. *Archives of Disease in Childhood* 2008;**93**:62–4. doi:10.1136/adc.2007.122531

26 Cole E, Lecky F, West A, *et al.* The impact of a pan-regional inclusive trauma system on quality of care. *Ann Surg* 2016;**264**:188–94. doi:10.1097/SLA.0000000000001393

27 Higginson I. Medical and practitioner staffing in emergency departments. London, UK: The College of Emergency Medicine 2015. https://www.rcem.ac.uk/docs/Workforce/RCEM%20Medical%20and%20Practitioner%20Staffing%20in%20EDs.pdf (accessed 6 Feb 2019).

28 Shah S. The real cost of emergency department physician turnover. Studer Group. 2016.https://www.studergroup.com/resources/articles-and-industry-updates/insights/january-2016/the-real-cost-of-emergency-department-physician-tu (accessed 12 Feb 2019).

29 Smith J, Keating L, Flowerdew L, *et al.* An emergency medicine research priority setting partnership to establish the top 10 research priorities in emergency medicine. *Emerg Med J* 2017;**34**:454–6. doi:10.1136/emermed-2017-206702

30 Addicott R, Maguire D, Honeyman M, *et al.* Workforce planning in the NHS. The King's Fund 2015.

FUNDED BY NIHR National Institute for Health Research

https://www.kingsfund.org.uk/sites/default/files/field/field_publication_file/Workforce-planning-NHS-Kings-Fund-Apr-15.pdf (accessed 6 Feb 2019).

31 College of Emergency Medicine. Emergency medicine consultants workforce recommendations. The College of Emergency Medicine 2010. https://www.rcem.ac.uk/docs/Workforce/CEM5324-Emergency-Medicine-Consultants---CEM-Workforce-Recommendations-Apr-2010.pdf (accessed 6 Feb 2019).

32 Heyworth J, Clancy M, Miles G. Emergency medicine operational handbook: the way ahead. London, UK: The College of Emergency Medicine 2011. https://www.rcem.ac.uk/docs/Policy/The%20Way%20Ahead_Final%20Dec%202011.pdf (accessed 6 Feb 2019).

33 Reynard K, Brown R. A clinical analysis of the emergency medicine workforce crisis. *Br J Hosp Med* 2014;**75**:612–6. doi:10.12968/hmed.2014.75.11.612

34 Thornton RJ, Skalnik P. *The early writings of Bronislaw Malinowski*. Cambridge, UK: Cambridge University Press 2006.

35 Becker HS. *Boys in white: student culture in medical school*. Piscataway, NJ: Transaction Publishers 2002.

36 Liberati EG, Tarrant C, Willars J, *et al.* How to be a very safe maternity unit: an ethnographic study. *Social Science & Medicine* 2019;**223**:64–72. doi:10.1016/j.socscimed.2019.01.035

37 LeCompte MD, Schensul JJ. *Designing & conducting ethnographic research: an introduction*. Lanham, MD: Rowman Altamira 2010.

38 Darbyshire D, Brewster L, Isba R, *et al.* Retention of doctors in emergency medicine: a scoping review protocol. *JBI Evidence Synthesis* 2020;**18**:154–162.

39 Sanday PR. The ethnographic paradigm(s). *Administrative Science Quarterly* 1979;**24**:527–38. doi:10.2307/2392359

40 Atkinson P. *Thinking ethnographically*. London, UK: SAGE 2017.

41 Grint K. *The sociology of work*. 4th ed. Cambridge, UK: Polity Press 2016.

42 Edgell S, Gottfried H, Granter E, *et al. The SAGE handbook of the sociology of work and employment*. 2016.

43 Hochschild AR. *The managed heart: commercialization of human feeling*. Berkerley, UNITED STATES: University of California Press 2012. http://ebookcentral.proquest.com/lib/lancaster/detail.action?docID=870020 (accessed 25 Jun 2019).

44 Woolcock M, Narayan D. Social capital: implications for development theory, research, and policy. *World Bank Res Obs* 2000;**15**:225–49. doi:10.1093/wbro/15.2.225

For peer review only

FUNDED BY NIHR | National Institute for Health Research

45 Slattery M. *Key ideas in sociology*. Oxford, UK: Nelson Thornes 2003.

46 Wacquant L, Akçaoğlu A. Practice and symbolic power in Bourdieu: the view from Berkeley. *Journal of Classical Sociology* 2017;**17**:55–69. doi:10.1177/1468795X16682145

47 Jafar AJN. What is positionality and should it be expressed in quantitative studies? *Emerg Med J* 2018;**35**:323–4. doi:10.1136/emermed-2017-207158

48 LeCompte MD, Goetz JP. Problems of reliability and validity in ethnographic research. Rev Educ Res 1982;**52**:31-60. doi:10.3102/00346543052001031

49 Rodgers BL, Cowles KV. The qualitative research audit trail: a complex collection of documentation. *Res Nurs Health* 1993;**16**:219–226.

50 Varpio L, Ajjawi R, Monrouxe LV, *et al.* Shedding the cobra effect: problematising thematic emergence, triangulation, saturation and member checking. *Med Educ* 2017;**51**:40–50. doi:10.1111/medu.13124

51 Given L, Saumure K. Trustworthiness. In: *The SAGE encyclopedia of qualitative research methods*. Thousand Oaks, CA: SAGE Publications 2008. doi:10.4135/9781412963909.n470

52 O'Reilly K. *Key concepts in ethnography*. Thousand Oaks, CA: SAGE 2008.

53 Pink S, Morgan J. Short-term ethnography: intense routes to knowing. *Symbolic Interaction* 2013;**36**:351–61. doi:10.1002/symb.66

54 Fetterman DM. *Ethnography: step by step*. 2nd ed. Thousand Oaks, CA: Sage Publications 1998.

55 Kvale S, Brinkmann S. *InterViews*. 3rd ed. London, UK: SAGE Publications 2014.

56 Silverman D. *Interpreting qualitative data*. 5th ed. London, UK: SAGE 2015.

57 QRS. *nVivo 12 Mac*. QRS International 2018.

58 NHS Data Dictionary. Accident and emergency department type. NHS Data Model and Dictionary Version 3. 2018.https://www.datadictionary.nhs.uk/data_dictionary/attributes/a/acc/accident_and_emergency_department_type_de.asp (accessed 5 Feb 2019).

59 Idelji-Tehrani S, Al-Jawad M. Exploring gendered leadership stereotypes in a shared leadership model in healthcare: a case study. *Medical Humanities* 2018;**45**:388–98. doi:10.1136/medhum-2018-011517

60 Brummell SP, Seymour J, Higginbottom G. Cardiopulmonary resuscitation decisions in the emergency department: An ethnography of tacit knowledge in practice. *Social Science & Medicine* 2016;**156**:47–54. doi:10.1016/j.socscimed.2016.03.022

For peer review only

FUNDED BY NIHR | National Institute for Health Research

61 Department for Constitutional Affairs. *Mental Capacity Act 2005: Code of Practice:* Norwich, UK: The Stationery Office 2007. https://assets.publishing.service.gov.uk/government/uploads/system/uploads/attachment_data/file/497253/Mental-capacity-act-code-of-practice.pdf (accessed 9 Jan 2020).

62 Murphy E, Dingwall R. Informed consent, anticipatory regulation and ethnographic practice. *Social Science & Medicine* 2007;**65**:2223–34. doi:10.1016/j.socscimed.2007.08.008

63 Braun V, Clarke V. Reflecting on reflexive thematic analysis. *Qual Res Sport Exerc Health* 2019;**11**:589–97. doi:10.1080/2159676X.2019.1628806

64 Clarke V, Braun V. Thematic analysis. In: Lyons E, Coyle A, eds. *Analysing qualitative data in psychology*. London, UK: SAGE 2016.

65 Braun V, Clarke V, Hayfield N, *et al.* Thematic analysis. In: Liamputtong P, ed. *Handbook of research methods in health social sciences*. Singapore: Springer Singapore 2019. 843–60. doi:10.1007/978-981-10-5251-4_103

66 Terry G. Doing Thematic Analysis. In: Lyons E, Coyle A, eds. *Analysing Qualitative Data in Psychology*. Thousand Oaks, CA: SAGE 2016.

67 Rodgers BL. Audit trail. In: *The SAGE encyclopedia of qualitative research methods*. Thousand Oaks, CA: SAGE Publications, Inc. 2008. doi:10.4135/9781412963909.n25

68 Hobbs D. Ethnography. In: Jupp V, ed. *The SAGE Dictionary of Social Research Methods*. London, UK: SAGE Publications Ltd 2006. https://methods.sagepub.com/reference/the-sage-dictionary-of-social-research-methods (accessed 18 Oct 2019).

69 Di Domenico M, Phillips N. Participant observation. In: *Encyclopedia of case study research*. Thousand Oaks, CA: SAGE Publications, Inc. 2010. doi:10.4135/9781412957397.n244

70 Rowe WE. Positionality. In: *The SAGE encyclopedia of action research*. Thousand Oaks, CA: SAGE Publications Ltd 2014. doi:10.4135/9781446294406.n277

71 May T, Perry B. *Reflexivity: the essential guide*. London, UK: SAGE 2017.

72 Bloor M, Wood F. *Keywords in qualitative methods*. London, UK: SAGE Publications Ltd 2006. doi:10.4135/9781849209403

For peer review only

FUNDED BY NIHR National Institute for Health Research

The James Lind Alliance

The James Lind Alliance (JLA) is a non-profit making initiative established in 2004. It brings patients, carers and clinicians together in Priority Setting Partnerships (PSPs) to identify and prioritise the Top 10 unanswered questions or evidence uncertainties that they agree are the most important.

The aim of this is to make sure that health research funders are aware of the issues that matter most to the people who need to use the research in their everyday lives. It is part of the NIHR (National Institute for Health Research), the research funding arm of the United Kingdom's NHS (National Health Service).

Figure 1. What is the James Lind Alliance? Information taken from the JLA website. http://www.jla.nihr.ac.uk/about-the-james-lind-alliance/ (accessed 15th January 2020

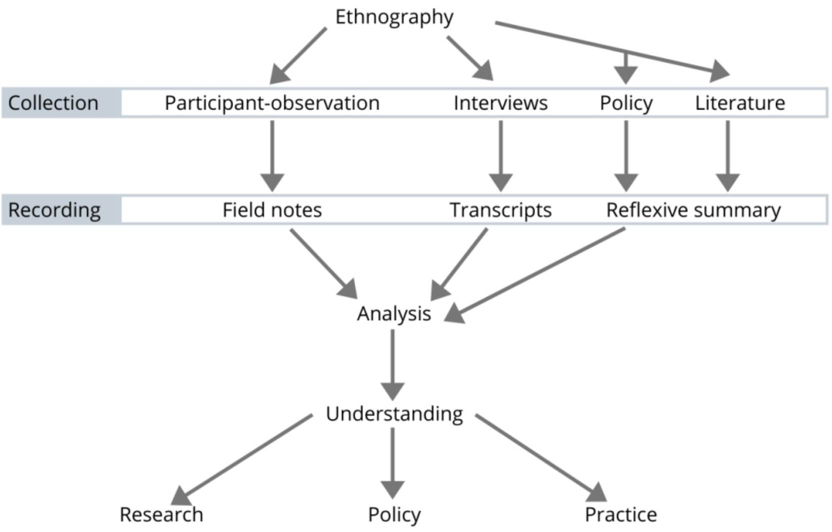

Figure 2. Summary of the ethnography

Lancaster University

Interview Guide

Where have all the doctors gone? An ethnographic study to explore the critical retention problem in Emergency Medicine

This guide will be used as a structure for interviews. It will allow the interviewer to draw out value from an interview and a structure to return to if the interview is going off topic. It will be shaped in response to specific observations in the fieldwork and tailored to the individual, with a focus on the overall research question: what makes doctors stay working in emergency medicine?

Pre-interview Checks

Ensure you have completed the consent procedure as outlined in the protocol.

Thank the participant for contributing.

Check the voice recording equipment is working.

Opening Question

To start the interview of ask some background questions, these will vary depending on your pre-existing knowledge of the participant.

- What is your role?
- How long have you worked in emergency medicine?
- What made you decide to work in emergency medicine?

This may direct the interview into retention, if not move onto directly asking about it.

What keeps you working in emergency medicine?

This is a very open question, follow up questions will depend upon the trajectory of the interview but may include:

Can you talk about a specific experience?
Prompts: what affected your decision? What happened after that experience?

Question Relating to Culture

How would you describe the culture of this emergency medicine department?
Is the culture here different to other EDs you have worked in?

For peer review only - http://bmjopen.bmj.com/site/about/guidelines.xhtml

Do you think that workplace culture affects staff retention?
How has it affected your work in emergency medicine?

Again, an open question, while generalities about retention and culture are interesting it is important as an interviewer to try and get to the personal experience.

Question Relating to Environment

How would you describe the work environment of this emergency medicine department?
Is the workplace here different to other EDs you have worked in?

How does the work environment affect your decision to work in EM?

Again, open questions, while generalities about retention and work environment are interesting it is important as an interviewer to try and get to the personal experience.

Digging for Depth

Having opened the conversation and grounded it in the interviewee's lived experience related to retention, the interviewer now needs to try and expand and develop any idea. Three tools are available for this:

1. *Being sensitive to ques from the previous questions and returning to them.*
2. *Asking about colleagues' experiences, building on specifics, and then returning to personal views about this.*
3. *Using a social media post to generate further discussion.*

This list is not exhaustive, nor it is a checklist. Some interviews may use all, some none, they are simply options that a skilled and attentive interviewer can apply to try and gain as much value from the interaction.

Post Interview Checklist

On completion of the interview a number of things need to be completed:

- Ensure the interviewee is alright, it is possible that the interview covered some difficult ground, the participant information sheet has sources of support.
- Thank participant for contributing.
- Are you ok? Remember the sources of support are available to the interviewer as well.
- Interviewees can be given a certificate of participation to include in their portfolio, taking part in research as a participant can help clinicians understand the process. This also acts as a formal thank you to participants.

Once the interviewee has vacated the interview space the interviewer should complete field notes related to the interview, the closer to the interview the better.

For peer review only - http://bmjopen.bmj.com/site/about/guidelines.xhtml

No.	Topic	Explanation and location
	**Title and abstract**	
S1	Title - Concise description of the nature and topic of the study Identifying the study as qualitative or indicating the approach or data collection methods	Title clearly states ethnographic approach, that this article is a study protocol and the problem of focus – retention of doctors in emergency medicine. Lines 4-5.
S2	Abstract - Summary of key elements of the study using the abstract format of the intended publication	Structured abstract appropriate to a protocol. Lines 22-48.
	**Introduction**	
S3	Problem formulation - Description and significance of the problem/phenomenon studied; review of relevant theory and empirical work; problem statement	Background and importance of problem outlined (lines 66-135), gap in the literature highlighted (lines 137-143).
S4	Purpose or research question - Purpose of the study and specific objectives or questions	Objectives and research questions clearly stated. Lines 156-163.
	**Methods**	
S5	Qualitative approach and research paradigm - Qualitative approach and guiding theory if appropriate; identifying the research paradigm is also recommended; rationale**	Ethnographic approach and possible theories outlined. Ethnographic paradigm highlighted. Lines 202-211.
S6	Researcher characteristics and reflexivity - Researchers' characteristics that may influence the research; potential or actual interaction between researchers' characteristics and the research	Highlighted as important in the protocol, will be detailed in the results. Lines 213-215.
S7	Context - Setting/site and salient contextual factors; rationale**	Site selection and rationale detailed. Lines 304-308.
S8	Sampling strategy - How and why research participants, documents, or events were selected; criteria for deciding when no further sampling was necessary (e.g., sampling saturation); rationale**	Sampling stratify for participant-observation and interview components detailed (lines 310-321). Discussion of theoretic saturation (lines 287-290).
S9	Ethical issues pertaining to human subjects - Documentation of approval by ethics review board and participant consent; other confidentiality and data security issues	Ethics approval documented (lines 417-419). Process of consent and capacity detailed (lines 323-382).
S10	Data collection methods - Types of data collected; details of data collection procedures including (as appropriate)	Plan to collect field notes (lines 262-263) and interview transcripts (lines 293-294) documented. Dates will be

For peer review only

	start and stop dates of data collection and analysis, iterative process, triangulation of sources/methods, and modification of procedures in response to evolving study findings; rationale**	included in report but are not stated in the protocol. Ethnographic studies are expected to evolve, how this occurs will be reported in the results but is not predicted in the protocol.
S11	Data collection instruments and technologies - Description of instruments and devices planned for data collection	Interview guides included as appendices. Use of voice recording software planned but not specified.
S12	Units of study - Number and relevant characteristics of participants, documents, or events included in the study; level of participation	Planned recruitment strategy outlined. Lines 284-286.
S13	Data processing - Methods for processing data prior to and during analysis, including transcription, data entry, data management and security, verification of data integrity, data coding, and anonymization/de-identification of excerpts	Recording and processing of field notes delineated. Interviews recorded and transcribed. Data stored and managed with nVivo 12 for Mac. Lines 293-295.
S14	Data analysis - Process by which inferences, themes, etc., were identified and developed, including the researchers involved in data analysis; usually references a specific paradigm or approach; rationale**	Data analysis plan, using reflexive thematic analysis, outlined. Lines 386-397.
S15	Techniques to enhance trustworthiness	Audit trail (lines 217-221), reflexivity and transferability discussed (lines 222-224).
	**Results/findings**	
S16	Synthesis and interpretation	Not predetermined in protocol.
S17	Links to empirical data	Not predetermined in protocol.
	**Discussions**	
S18	Integration with prior work, implications, transferability, and contribution(s) to the	Not predetermined in protocol.
S19	Limitations - Trustworthiness and limitations of findings	Anticipated limitations discussed. Lines 404-413.
	**Other**	
S20	Conflicts of interest - Potential sources of influence or perceived influence on study conduct and conclusions; how these were managed	Lines 459-461.
S21	Funding - Sources of funding and other support; role of funders in data collection, interpretation, and reporting	Lines 465-467.

For peer review only

Table 1. SRQR Table - Standards for Reporting Qualitative Research. The SRQR tool is designed for reporting of completed studies, as such certain aspects are merely acknowledged are requiring inclusion in the final report. The table allows the reader to map the study protocol to a recognised quality indicator. The topic descriptions have been maintained, but edited for brevity, the full description can be found in the original publication.[1]

For peer review only

BMJ Open

‘Where have all the doctors gone?’ A protocol for an ethnographic study of the retention problem in Emergency Medicine

Journal:	*BMJ Open*
Manuscript ID	bmjopen-2020-038229.R1
Article Type:	Protocol
Date Submitted by the Author:	23-Jun-2020
Complete List of Authors:	Darbyshire, Daniel; Lancaster University Faculty of Health and Medicine, Medical School; Salford Royal NHS Foundation Trust, Emergency Department<br>Brewster, Liz; Lancaster University, Lancaster Medical School<br>Isba, Rachel; Lancaster University Faculty of Health and Medicine, Medical School; North Manchester General Hospital, Children's Accident and Emergency Department<br>Body, Richard; The University of Manchester, Division of Cardiovascular Sciences; Manchester University NHS Foundation Trust, Emergency Department<br>Goodwin, Dawn; Lancaster University Faculty of Health and Medicine, Medical School
**Primary Subject Heading**:	Emergency medicine
Secondary Subject Heading:	Health services research, Health policy, Medical education and training, Qualitative research, Sociology
Keywords:	ACCIDENT & EMERGENCY MEDICINE, Health policy < HEALTH SERVICES ADMINISTRATION & MANAGEMENT, MEDICAL EDUCATION & TRAINING, QUALITATIVE RESEARCH

SCHOLARONE™
Manuscripts

I, the Submitting Author has the right to grant and does grant on behalf of all authors of the Work (as defined in the below author licence), an exclusive licence and/or a non-exclusive licence for contributions from authors who are: i) UK Crown employees; ii) where BMJ has agreed a CC-BY licence shall apply, and/or iii) in accordance with the terms applicable for US Federal Government officers or employees acting as part of their official duties; on a worldwide, perpetual, irrevocable, royalty-free basis to BMJ Publishing Group Ltd ("BMJ") its licensees and where the relevant Journal is co-owned by BMJ to the co-owners of the Journal, to publish the Work in this journal and any other BMJ products and to exploit all rights, as set out in our licence.

The Submitting Author accepts and understands that any supply made under these terms is made by BMJ to the Submitting Author unless you are acting as an employee on behalf of your employer or a postgraduate student of an affiliated institution which is paying any applicable article publishing charge ("APC") for Open Access articles. Where the Submitting Author wishes to make the Work available on an Open Access basis (and intends to pay the relevant APC), the terms of reuse of such Open Access shall be governed by a Creative Commons licence – details of these licences and which Creative Commons licence will apply to this Work are set out in our licence referred to above.

Other than as permitted in any relevant BMJ Author's Self Archiving Policies, I confirm this Work has not been accepted for publication elsewhere, is not being considered for publication elsewhere and does not duplicate material already published. I confirm all authors consent to publication of this Work and authorise the granting of this licence.

For peer review only

Title Page

Title

‘Where have all the doctors gone?’ A protocol for an ethnographic study of the retention problem in Emergency Medicine

Corresponding author: Dr Daniel Darbyshire, B32 Furness College, Lancaster University, Bailrigg, LA1 4YG. d.darbyshire@lancaster.ac.uk (+44)0 7757119141

Authors and affiliations

Daniel Darbyshire (1,2), Liz Brewster (1), Rachel Isba (1,3), Richard Body (4,5), Dawn Goodwin (1)

(1) Lancaster Medical School, Bailrigg, UK. (2) Emergency Department, Salford Royal Hospital NHS Foundation Trust, Salford, UK. (3) Children’s Accident and Emergency Department, North Manchester General Hospital, Manchester, UK. (4) Division of Cardiovascular Sciences, The University of Manchester, Manchester, UK. (5) Emergency Department, Manchester University NHS Foundation Trust, Manchester, UK

Word count = 3978

Keywords - Ethnography, staffing, emergency medicine, sustainability.

Abstract

Introduction

‘Emergency medicine (EM) in the United Kingdom (UK) has a medical staffing crisis.’ Inadequate staffing, in EM and across healthcare, is a problem that affects the quality of patient care globally. Retention of doctors in EM is a particularly acute problem in the UK’s National Health Service (NHS). Sustainable careers in healthcare are gaining increasing attention at a national and international policy level, but research to understand the factors that facilitate retention is lacking.

This study aims to develop understanding of what drives retention of doctors in EM by focusing on those who remain in these careers, where previous research has targeted those who have left. By addressing the problem of retention in a different way, using innovative methods in this context, we aim to develop a deeper and more nuanced understanding of sustainable careers in EM.

Methods and analysis

This is an ethnographic study combining participant observation in two emergency departments, interviews with doctors from these departments, from organisations with influence or interest at a policy level and with doctors who have left emergency medicine. The analyses will integrate detailed workplace observation alongside key academic and policy documents using reflexive thematic analysis.

Ethics and dissemination.

Approvals have been obtained from Lancaster University via the Faculty of Health and Medicine Research Ethics Committee (FHMREC18058) and the Health Research Authority (IRAS number 256306). The findings will inform understanding of sustainable careers in EM

For peer review only - http://bmjopen.bmj.com/site/about/guidelines.xhtml

that may be transferable to other settings, professions, and locations that share key characteristics with EM such as paediatrics, emergency nursing, and general practice. Findings will be disseminated through a series of academic publications and presentations, through local and specialty research engagement, and through targeted policy statements.

Strengths and Weaknesses

- Focuses on a national research priority and a global problem.
- Takes a novel approach to understanding the retention problem.
- Engaged with policy makers and the public throughout the research.
- Some aspects of retention will vary by context.
- Policies and practice will evolve during the research, making it harder to unpick some of the findings.

For peer review only

FUNDED BY NIHR | National Institute for Health Research

Main Text

Main text separated under appropriate headings and subheadings using the following hierarchy: BOLD CAPS, bold lower case, Plain text, Italics

INTRODUCTION

The specialty of emergency medicine (EM) provides care across the spectrum of illness and injury to people of all ages. It operates all day, every day and in the United Kingdom (UK) has seen demand increase year on year, with declining performance in terms of patients having their emergency department (ED) visit concluded within four hours, and a rise in numbers of patients spending greater than 12 hours in the ED.[1] Much of this declining performance is attributed to factors outside of the ED, with exit block due to insufficient hospital beds and community services being the most frequently cited contributing factors.[2,3] Arguably, the most important factor for high-quality care in the ED that departments have some control over, is staffing.

Unfortunately, 'Emergency Medicine in the UK has a medical staffing crisis.'[4] Starting with the formation of the Emergency Medicine Taskforce, set up in 2011,[4] the specialty has sought to change practice, policy and culture though innovations such as the 'less than full time training' pilot,[5] increasing recognition of the particular stresses a career in EM brings and a focus on sustainable careers.[6,7] This has led to EM growing rapidly in terms of both consultant and trainee numbers but demand for care has outstripped staffing growth and looks to continue to do so.[8]

The EM workforce problem is occurring in the context of staffing shortages in much of the United Kingdom's National Health Service (NHS)[9] and on a background of demand outstripping growth in the global healthcare workforce.[10] The picture is complex. The NHS employs around one million people, with the workforce increasing by around 1.8% from July 2017 to 2018. However, organisations report collective vacancies across the NHS in the region of 100,000 and falling numbers of key staff groups.[9] The *NHS Long Term Plan*,[11] published in January 2019 (updated August 2019), followed by the *Interim People Plan*,[12] outlined the UK Government's proposed solutions for staffing the NHS. The plans – which focused on staff development, empowerment, and improving leadership culture – were broadly welcomed.[13] There were, however, concerns, for example about the time lag between increasing training numbers and generating qualified practitioners in employment, with potential over-reliance on international recruitment in the interim.[13,14]

From recruitment to retention

EM historically had a recruitment problem, but after much work by the then College of Emergency Medicine (now the Royal College of Emergency Medicine) and Health Education England, amongst others, in improving the image of the specialty and the experience of trainees, fill rates for EM training posts reached 96% in 2011.[15] Recruitment has since fallen from the 2011 high, yet it remains strong and competitive, when compared with other specialities, at 86% in 2019 (compared to 77% for internal medicine and 69% for paediatrics).[16] However, attrition from training programmes is unsustainably high, with The King's Fund (an independent English think tank) reporting that: 'problems with retention mean it [emergency medicine] has the greatest attrition rate of any medical

FUNDED BY NIHR National Institute for Health Research

For peer review only

specialty, with almost 50 per cent of registrar doctors in their third or fourth year of training resigning.'[17]

Following on from a 2014 survey of trainees that looked at the attractiveness of a career in EM,[18] the Emergency Medicine Trainees' Association now conduct its own annual surveys.[19–21] These surveys elucidate the challenges faced by trainees, specifically burnout, poor working conditions, and workload pressures. This is supported by the General Medical Council's national training survey which showed EM as the specialty with the highest work intensity[22] and by the high attrition from the emergency medicine specialty training programme.[23] The data on trainees is reflected for consultants and staff and associate specialist grades, with all groups being undersubscribed and experiencing exodus.[23]

Recruiting and retaining EM clinicians is fundamentally connected to the provision of high-quality clinical care. More experienced and senior doctors deliver better care, performing fewer unnecessary tests and getting fewer complaints, and experience is only gained by retaining doctors within the specialty.[24–26] Retention of doctors affects wider health and care services in two main ways. Firstly, the presence of doctors with more experience results in efficiency savings both in the ED and downstream in a patient's hospital journey, with recent studies finding a greater than 20% reduction in admission rates, reduced waiting times, and cost savings of £3 million to one hospital.[24,25] The introduction of the Major Trauma Networks in 2010 with consultant-led care of severely injured patients in London saved an estimated 58 lives in the first year.[26] Secondly, staff turnover is costly and disruptive. When ED staff leave it is highly unusual for replacements to be found quickly, if at all, often leading to locum (temporary) doctors filling in who are 'generally less efficient, and less safe, than permanent members of staff'.[27] Costs in the UK are not clear but an approximate figure for replacing one fully trained emergency physician in the US is $164,000.[28]

We would argue that the evidence is therefore clear that staff retention is important for quality emergency care. What is not clear is what drives retention and what allows people to have sustainable careers. This knowledge gap was also identified in the 2017 James Lind Alliance (see figure 1) priority setting partnership for EM research. Number four in their list of the top ten research priorities in EM asked: 'with regard to how ED staff development is managed, what initiatives can improve staff engagement, resilience, retention, satisfaction, individuality and responsibility?'[29] This priority was supported by calls from various other stakeholders to address the issue of retention including The King's Fund,[17,30] Health Education England[15], and the Royal College of Emergency Medicine.[27, 31–33]

The proposed study aims to address retention of doctors in EM – an innovative approach, contrasting with previous work that has focused predominantly on why people leave. The ethnographic methodology is also innovative in this setting as the retention problem has not previously been explored using this approach. By developing fresh insights into the complex and interacting factors that positively influence retention, we hope to inform future efforts aimed at reducing exodus from the specialty, at both a research and policy level.

Research aim and objectives

FUNDED BY NIHR | National Institute for Health Research

For peer review only - http://bmjopen.bmj.com/site/about/guidelines.xhtml

The aim of this study is to gain a deep understanding of retention in EM, in order to elucidate how retention is made possible. This aim requires a study design that can tackle complexity and develop new understandings. The objectives are to:

- understand in detail the day-to-day lived experience of EM doctors, to identify and explore factors influencing retention;
- situate these descriptions within the current educational and health policy contexts;
- advance the debate and make policy and practice recommendations based on a detailed understanding of retention of doctors in EM.

METHODS

Overview

We will conduct an ethnographic study of EM doctors in two type 1 EDs in the North West of England. Ethnography is derived from anthropology and social sciences and is historically associated with gaining cultural understandings about groups, with the origins of the practice going back to colonial-era studies of 'exotic' civilisations.[34] Its use within health research is broad and well-established, from a 1961 study of medical student culture[35] through to a recent study trying to understand why one particular maternity unit performs highly.[36]

Ethnography is 'a systematic approach to learning about social and cultural life of communities, institutions, and other settings'.[37] Key features of ethnography relevant to this study are that it is investigative, the primary tool of data collection is the researcher, and that it 'emphasises and builds on the perspective of the people in the research setting'.[37]

Ethnography creates understanding through description and analysis. This usually involves going to a place and collecting data – which is often a detailed description of what happens in that place along with the accompanying talk – over a period of time. What information is recorded, how long the researcher spends there, and what other forms of data are collected, depends on the context of the research and question being asked. For this research, ethnography refers to the totality of the study including the time spent observing in the department (referred to as participant-observation hereafter), interviews, and policy documents.

This ethnographic study will involve the lead researcher spending time in two different EDs, trying to understand what allows doctors to have sustainable careers. This will involve observing the people, space, and happenings in the departments, and speaking to those in these spaces, keeping the focus on retention. This will be supported by interviews with doctors (conducted away from the department) and with others who can inform the research aim and objectives, and by critical review of policy and academic literature relating to retention. The literature search strategy, in the form of a scoping review, is described elsewhere.[38] Each step in the process is expanded on below and summarised in figure 2.

This study subscribes to the 'ethnographic paradigm'[39], by this we mean that we don't see value in predetermining a particular way of seeing the world, and agree with Atkinson when he writes that 'ideas can and should come from a variety of sources'.[40] This study draws

For peer review only

For peer review only - http://bmjopen.bmj.com/site/about/guidelines.xhtml

on the sociological practice of ethnography and as such the findings will be analysed, interpreted, and challenged through a number of sociological theories. These will include, but are not limited to, theories of work[41,42] such emotional labour[43] and social capital,[44] theories of community such as Tönnies discussion of Gemeinschaft and Gesellschaft (community and society)[45], and Bourdieu's conception of symbolic power.[46] This list is not intended to be prescriptive – it aims to show theories that may prove informative.

As the study is built on the interpretation of DD (the researcher collecting the data and performing the analysis), a discussion of their positionality will be included when the study reports.[47] This is an integral part of reflexive thematic analysis described below, 'addressing threats to credibility in ethnography requires different techniques from those used in experimental studies.'[48] Our study aims to produce a clear audit trail, of which publishing this protocol is a part.[38,49] Other important parts of the audit trail are notes made during observations about why the researcher has chosen to observe at particular times and locations, documents generated around meetings of the research team, and documents from peer-review, ethics, and the funder which have shaped the study.[49] We are aiming for transferability not generalisability, reflecting the importance of context,[50] and will utilise reflexive thematic analysis, sense checking from the public involvement group. We aim for confirmability by aligning interpretations and data in the final report.[51]

Patient and public involvement

The public has been involved in the development of this study at several levels and at different key points in the development of the protocol. The aforementioned James Lind Alliance priority setting exercise demonstrated the public's belief that the topic areas is important.[29] To confirm this, with the support of the NIHR Research Design Service North West and the Research and Development Department at Blackpool Victoria Hospital, we ran a public involvement event. During this event, members of the public agreed with the James Lind Alliance that exploring factors related to the retention of doctors in EM merited investigation. Participants thought the proposed methods were acceptable to patients, but questioned whether ethnography would be seen as acceptable to a clinical audience. A member of the initial patient involvement group has volunteered to be further involved in the study by becoming a member of the steering committee and by reviewing the conclusions we gather during the scoping review part of the study.[38]

As the main focus of the study is EM doctors, we also sought their views as part of our engagement strategy. We presented the research plan, along with some of the preliminary findings from the scoping review,[38] at the Emergency Medicine Trainees' Association National Conference in Cardiff, in November 2018. Feedback was obtained during the question and answer question and by providing a link to an online form. This process was repeated at the Oxford Society for Emergency Medicine Conference and the Association for the Study of Medical Education Researching Medical Education Meeting in London, both in December 2018. Thirteen attendees provided written feedback, including one offer to help, which led to recruitment of a second reviewer for the scoping review, with many more providing verbal comments and questions. Including interviews with doctors who have left was suggested during this professional engagement, and again during peer review from the NIHR when funding was being obtained, and subsequently included in the research plan.

FUNDED BY NIHR National Institute for Health Research

Participant-observation

Participant-observation refers to the time spent in the research setting watching, listening, and documenting. It is strongly linked with ethnography and is sometimes synonymous with field work. These terms are contentious[52] and for clarity we refer to participant-observation as the component of ethnography that occurs in the research setting. All data collection will be conducted by DD who will undertake observation at two sites, each for a period of twelve weeks. At each site they will perform observations in all parts of the department and at all times of the day. A day of participant-observation will be divided in two. The first half of the day will be spent conducting observations and writing field notes, incorporate talking with staff and documenting what they say, and the researcher's thoughts about the encounter. We will investigate the working environment, interaction between staff, and behaviours related to retention. The field notes will be handwritten in a notebook. The other half of the day will involve typing up, reflecting on, and expanding field notes. We aim to perform four days of observation each week by scheduling forty-eight days over the twelve-week period with start times reflecting the shift patterns locally, but to include days, evening, nights and weekends. A total of twenty-four weeks' participant-observation was chosen as it will allow DD to participate in observations at all times, with flexibility to repeat observations at key times or with specific key informants. Traditionally ethnographic observation has been conducted over a much greater period of time than the one that proposed, to allow for a period of familiarisation. This process will be accelerated in this study due to DD's pre-existing knowledge of EM and EDs (as a senior trainee in EM). This more focused form of ethnography has a developing literature for its use in complex healthcare settings[53] and based on this work, and the intensity of the observation within the twelve-week period, it is estimated that this will be sufficient to meet the aims of the study.

Interviews

In addition to the participant-observation conversations described above, we will conduct interviews, reserving the term for the physically separate, planned, audio-recorded and transcribed encounters. DD will conduct all the interviews, aiming to complete around 40. Ten interviews with doctors across all levels of seniority from each of the two research sites, ten interviews with individuals within key organisations relevant to retention in EM (such as the Royal College of Emergency Medicine, the Emergency Medicine Trainees' Association, Health Education England, and NHS Improvement), and ten with doctors who have left EM. Potential participants for interviews will be identified during the periods of participant-observation and the literature review and supplemented by recommendations obtained from interviewees. Interviewing ten doctors from each research site would represent between a quarter and a third of doctors at each level present on a rota at any time. The sample size has been agreed upon by discussion within the research team, who have extensive experience with this methodology, and by consulting key texts, and represents an estimation for when theoretical saturation (see glossary) might be achieved.[54–56]

Interviews will take place in a neutral space which allows for private conversation. The interview guide is available in Appendix 1. However, it is expected to evolve as the study progresses, based on pilot interviews, and ongoing analysis. Interviews will be digitally recorded, transcribed, and imported into nVivo 12,[57] the qualitative data analysis computer

FUNDED BY NIHR | National Institute for Health Research

For peer review only - http://bmjopen.bmj.com/site/about/guidelines.xhtml

package that facilitates the organisation and analysis of research data. After each interview DD will record reflections in the field notes.

Policy and literature

Policy documents (grey literature) will be identified through the aforementioned scoping review[38] and from the other aspects of the ethnography. Key documents will be chosen based on relevance to research context and incorporated into the analysis. A similar process will be used for academic literature.

Inclusion criteria

Participant observation sites were selected based on the following factors:

- presence of foundation, core, and higher trainees;
- presence of staff and associate specialists;
- Type 1 ED (major ED, providing a consultant-led 24-hour service with full resuscitation facilities).[58]

Interview and observation candidates

Within the two EDs the focus for participant observation, and interview candidates is:

- doctor;
- working in the study site;
- staff member for at least 4 weeks
- aim to include a maximally inclusive representation of the department, including age, gender, seniority, international medical graduates, and UK trained doctors.

The inclusion criteria are intended to be broad and inclusive. The 4-week criteria were chosen to avoid interviewing very new staff members at the start of their work. The longitudinal nature of the ethnography means that these candidates may be eligible once they have been in the department for a month or more.

Consent

Participant-observation

Informed consent needs to match the research and the environment in which it is being undertaken. We are not alone in making this point, several previous studies within the NHS[36,59] and the ED[60] have taken a proportionate route, clearly demonstrating the acceptability and utility of such an approach.

To ensure participants are informed of the study protocol, aims, and data collection methods, a continuous information sharing approach will be taken, mirroring the studies referenced above. The research has already been explained to the senior leadership at each study site, along with the research and development leads. To complement this, we will present the research plan to the medical and nursing staff at scheduled meetings such as junior doctors teaching, band 5, 6 and 7 nursing meetings, consultant meetings and departmental research and governance meetings. We will advertise the start of the study with posters giving people the opportunity to ask questions, and state objections, before it starts. Emails will be circulated to all ED staff informing them of the study. In addition, we will offer a brief explanation to staff members who the researcher interacts with in the department, as both part of the consent process and as a way to start conversations as part of the research method.

FUNDED BY NIHR National Institute for Health Research

When introducing himself in the research setting DD will reiterate the voluntary nature of the study and that it is ok for them to ask for observation to stop, either temporarily or until further notice. Based on previous studies in the ED this is unlikely to occur frequently, and as such we intend, if this happens, to relocate to a different part of the department to continue observation. If the objection is not time limited then the researcher would not attend when that person was on shift, the 24-hour nature of EM makes this feasible. Should an individual ask for more information about the study we will provide them with a short summary of the study, a link to this protocol and contact details for the research team.

Patients are not participants in this study, and no patient identifiable data will be recorded, but they, and other staff members, do occupy the research space. Their confidentiality and acceptance of the research is vital. We believe it is unfeasible, overly burdensome, and unnecessary to obtain consent from patients transiting through the ED. Either DD will introduce themselves or will be introduced by the member of staff they are accompanying. If a patient does not want to have the care they receive observed, DD will withdraw from that episode of care. DD will rely on his experience and expertise of working within this environment to proportionally address this issue in real time.

Informed consent for ethnographic study is different to other types of research, particularly so in unpredictable settings such as the ED. The use of the standard written consent procedure is overly burdensome to the researcher and participant, not feasible, and would bar patients and staff members who occupy these spaces from benefiting from this type of research.[61]

Interviews

Formal written consent, supported by a participant information sheet, will be obtained for the formal, recorded, interviews. Consent will be obtained by DD prior to conducting the interview. Having expressed initial interest in participating, potential interviewees will be provided with a copy of the consent form and participant information sheet. On the day of the interview, the interviewee will be given another opportunity to review the participant information sheet and consent form, with DD on hand to answer any questions. Some interviewees, especially those with high-profile roles in national organisations, may feel that anonymisation is either impossible or not in their interests; they will be given the option of participating in the study in a non-anonymised fashion. They will retain the right to have their interview anonymised, and all interviewees have the right to withdraw, up to the point of publication.

ANALYSIS

During the data collection phase of the study, initial analysis will take the form of reflective analytical notes recorded in the fieldnotes, these observations and interpretations guide the ongoing analysis. Once data collection is complete, a more thorough, systematic analysis of fieldnotes, interview transcripts and literature and policy documents will be completed utilising reflexive thematic analysis.[62–64] This process leads to generation of themes – these are not groups of data that are about the same thing, but ‘stories about particular patterns of shared meaning across the dataset’.[62] Reflexive thematic analysis emphasises

For peer review only

FUNDED BY NIHR National Institute for Health Research

the effortful nature of analysis, in that themes are not found within the data, rather they are created though analytical work. This highlights the role of the researcher in generating the analysis, laying this open to scrutiny. The analysis will use the six-stage approach to thematic analysis (see Figure 3 for a summary).[62,65] These stages are descriptive and supportive, as opposed to having clear divisions. It is expected that the analysis will go back and forth through the stages multiple times.

Limitations

The study is geographically limited to the North West of England. Much of the activity of EDs is similar nationally, but local differences will mean the findings will require interpretation and translation to other locales.

The study is based at two sites. There is no reliable objective way of stating if the sites have high or low retention, relative to the national picture. If they are extreme outliers it may be difficult for other sites to interpret the findings.

This study is focused solely on doctors working in EDs, there may be some transferability to other professions or working environments, but this is a limitation.

Data collection by someone without experience of the ED may lead to different conclusions, though this might be at the expense of access problems and detailed understanding of the work in the ED.[52]

ETHICS AND DISSEMINATION

Approval from Lancaster University via the Faculty of Health and Medicine Research Ethics Committee (FHMREC18058) was received on 15th April 2019. Health Research Authority approval was formalised on 11th November 2019, IRAS number 256306. Traditional academic dissemination will occur locally through seminars and lectures, and to a broader audience though conference presentations and academic publications. Dissemination to the public will be facilitated by presentations at knowledge translation events and by engaging with local media and research translation services such as The Conversation. Policy briefs summarising the findings will be provided for key audiences such as NHS England, Health Education England, and the Royal College of Emergency Medicine.

Glossary

Audit trail – a thorough collection of documentation regarding all aspects of the research. Qualitative research often evolves though the iterative process of data collection and analysis, requiring the researcher to make frequent decisions which alter the course of the research. Documents that form this trail of evidence include field notes, a research diary, and correspondence.[49,66]

Ethnography – a method based on description and the key assumption that ‘personal engagement with the subject is the key to understanding a particular culture or social

For peer review only

FUNDED BY NIHR National Institute for Health Research

setting'.[67] It can involve many different ways of collecting data, but is strongly associated with participant-observation and field work.

Participant-observation – a combination of a wide variety of methods including observation, informal interviews and conversations, combined with analysis of other materials encountered in the research setting.[68]

Positionality – how the researcher is positioned with respect to the social and political context of the study. Affects all aspects from formation of the research question to analysis. Making this explicit allows the reader to interpret the findings in context.[47,69]

Reflexivity – conscious examination and explanation of how the researcher's positionality influences and is influenced by the research as it progresses.[70]

Theoretical saturation – the continuation of sampling and data collection until no new conceptual insights are generated. At this point the researcher has provided repeated evidence for his or her conceptual categories.[71]

Trustworthiness – the ways in which a qualitative researcher ensures quality in their research, specifically in terms of transferability and credibility.[51] Moves discussions about research quality away from the quantitative paradigm of triangulation and generalisability.

Competing interests statement

This publication presents independent research funded by the National Institute for Health Research (NIHR). The views expressed are those of the author(s) and not necessarily those of the NHS, the NIHR or the Department of Health and Social Care.

All authors have completed the ICMJE uniform disclosure form at www.icmje.org/coi_disclosure.pdf and declare: DD had financial support from the NIHR, BMA Foundation and the Royal College of Emergency Medicine for the submitted work; no financial relationships with any organisations that might have an interest in the submitted work in the previous three years; no other relationships or activities that could appear to have influenced the submitted work.

Funding statement

Daniel Darbyshire is funded by a National Institute for Health Research (NIHR) Doctoral Fellowship for this research project. Initiation of the study was supported by the BMA Foundation Kathleen Harper Award and the RCEM Young Investigator Award.

Author statement

DD conceived the project. DD, LB, RB and DG developed the initial proposals for funding. DD, LB, DG, RI and RB developed the protocol. DD produced the initial draft of the manuscript. LB and DG are providing direct PhD supervision for DD. RB and RI are supporting the PhD with specialty and institutional expertise respectively. All authors contributed to and revised the final manuscript.

Acknowledgements

FUNDED BY NIHR | National Institute for Health Research

For peer review only

The authors would like to acknowledge the ongoing work of Gavin Quick and Anne Clark for their public and patient involvement role in developing the protocol.

References

1 Royal College of Emergency Medicine. RCEM Winter flow project: final report July 2019. London, UK: Royal College of Emergency Medicine. 2019. https://rcem.ac.uk/docs/RCEM_Winter_Flow_Project_Final_Report_2019.pdf (accessed 11 Oct 2019).

2 Mason S, Knowles E, Boyle A. Exit block in emergency departments: a rapid evidence review. *Emerg Med J* 2017;**34**:46–51. doi:10.1136/emermed-2015-205201

3 Knowles E, Mason SM, Smith C. Factors associated with exit block and impact on the emergency department. *Emerg Med J* 2017;**34**:61–2. doi:10.1136/emermed-2015-205202

4 Hughes G. The emergency medicine taskforce: an interim report. *Emerg Med J* 2013;**30**:348–348. doi:10.1136/emermed-2012-202260

5 Clancy M. The Less Than Full Time (LTFT(3)) working in emergency medicine pilot final report. London, UK: The Royal College of Emergency Medicine. 2019. https://www.rcem.ac.uk/docs/Training/LTFT_pilot_FINAL_REPORT-for_website.pdf (accessed 11 Oct 2019).

6 Hewitt S, Kennedy U. EM-POWER: a wellness compendium for EM. London, UK: The Royal College of Emergency Medicine. 2019. https://www.rcem.ac.uk/docs/Sustainable%20Working/0.%20Wellness%20Compendium%20(Apr%202019).pdf (accessed 11 Oct 2019).

7 RCEM Sustainable Working Practices Committee. EMPOWER: A guide to engage and retain your established EM staff. London, UK: Royal College of Emergency Medicine. 2018. https://www.rcem.ac.uk/docs/Workforce/Engage%20and%20Retain%20Sept%202018.pdf (accessed 20 Aug 2019).

8 Smith E, RCEM Service Design and Configuration Committee. RCEM workforce recommendations 2018: consultant staffing in emergency departments in the UK. London, UK: The Royal College of Emergency Medicine. 2018. https://www.rcem.ac.uk/docs/Workforce/RCEM%20Workforce%20Recommendations%202018%20-%20Consultant%20Staffing.pdf (accessed 6 Feb 2019).

9 Buchan J, Charlesworth A, Gershlick B, *et al.* A critical moment: NHS staffing trends, retention and attrition. London, UK: The Health Foundation. 2019. https://www.health.org.uk/sites/default/files/upload/publications/2019/A%20Critical%20Moment_1.pdf (accessed 11 Oct 2019).

For peer review only

10 Britnell M. Human: Solving the global workforce crisis in health care. The Nuffield Trust. 2019. https://www.nuffieldtrust.org.uk/news-item/human-solving-the-global-workforce-crisis-in-health-care (accessed 10 Jun 2019).

11 NHS England. The NHS long term plan. London, UK: NHS. 2019. https://www.longtermplan.nhs.uk/wp-content/uploads/2019/08/nhs-long-term-plan-version-1.2.pdf (accessed 11 Oct 2019).

12 NHS Improvement. Interim NHS people plan. London, UK: NHS Improvement. 2019. https://improvement.nhs.uk/resources/interim-nhs-people-plan/ (accessed 10 Jun 2019).

13 Bailey S. The King's Fund responds to the interim NHS people plan. London, UK: The King's Fund. 2019. https://www.kingsfund.org.uk/press/press-releases/interim-nhs-people-plan (accessed 11 Oct 2019).

14 Campbell J, Dussault G, Buchan J, *et al.* A universal truth: no health without a workforce. Geneva, Switzerland: Global Health Workforce Alliance and World Health Organization. 2013. https://www.who.int/workforcealliance/knowledge/resources/GHWA-a_universal_truth_report.pdf (accessed 10 Jun 2019).

15 Health Education England. Emergency medicine: background to HEE proposals to address workforce shortages. London, UK: Health Education England. 2013. https://www.hee.nhs.uk/our-work/emergency-medicine (accessed 6 Feb 2019).

16 Health Education England. Specialty recruitment: round 1 - acceptance and fill rate. London, UK: Health Education England. 2018.https://www.hee.nhs.uk/our-work/medical-recruitment/specialty-recruitment-round-1-acceptance-fill-rate (accessed 9 Aug 2019).

17 The King's Fund. What's going on in A&E? London, UK: The King's Fund. 2014. https://www.kingsfund.org.uk/projects/urgent-emergency-care/urgentand- emergency-care-mythbusters (accessed 5 Feb 2019).

18 Joy T, Moran J, Hassan T, *et al.* The attractiveness of a career in Emergency Medicine: A survey of UK trainees. London, UK: The College of Emergency Medicine. 2014. http://www.emtraineesassociation.co.uk/emta-surveys.html# (accessed 5 Feb 2019).

19 Archer K, Bailey J, Jenkinson E. EMTA trainee survey 2015. London, UK: Emergency Medicine Trainees Association. 2016. http://www.emtraineesassociation.co.uk/emta-surveys.html# (accessed 4 Feb 2019).

20 Bailey J, Archer K, Stewart P, *et al.* EMTA survey 2016. London, UK: Emergency Medicine Trainees Association 2017. http://www.emtraineesassociation.co.uk/emta-surveys.html# (accessed 4 Feb 2019).

21 Bailey J, Mashru A, Stewart P, *et al.* EMTA survey 2017. London, UK: Emergency Medicine Trainees Association. 2018. http://www.emtraineesassociation.co.uk/emta-surveys.html# (accessed 4 Feb 2019).

FUNDED BY NIHR National Institute for Health Research

22 General Medical Council. National training survey 2019: Initial findings report. Manchester, UK: GMC 2019. https://www.gmc-uk.org/-/media/national-training-surveys-initial-findings-report-20190705_2.pdf?la=en (accessed 11 Oct 2019).

23 Health Education England, NHS England, NHS Improvement, *et al.* Securing the future workforce for emergency department in England. London, UK: NHS Improvement. 2017. https://improvement.nhs.uk/documents/1826/Emergency_department_workforce_plan_-_111017_Final.3.pdf (accessed 28 May 2019).

24 White AL, Armstrong PAR, Thakore S. Impact of senior clinical review on patient disposition from the emergency department. *Emergency Medicine Journal* 2010;**27**:262–5. doi:10.1136/emj.2009.077842

25 Geelhoed GC, Geelhoed EA. Positive impact of increased number of emergency consultants. *Arch Dis Child* 2008;**93**:62–4. doi:10.1136/adc.2007.122531

26 Cole E, Lecky F, West A, *et al.* The impact of a pan-regional inclusive trauma system on quality of care. *Ann Surg* 2016;**264**:188–94. doi:10.1097/SLA.0000000000001393

27 Higginson I. Medical and practitioner staffing in emergency departments. London, UK: The College of Emergency Medicine. 2015. https://www.rcem.ac.uk/docs/Workforce/RCEM%20Medical%20and%20Practitioner%20Staffing%20in%20EDs.pdf (accessed 6 Feb 2019).

28 Shah S. The real cost of emergency department physician turnover. Pensacola, FL,: Studer Group. 2016. https://www.studergroup.com/resources/articles-and-industry-updates/insights/january-2016/the-real-cost-of-emergency-department-physician-tu (accessed 12 Feb 2019).

29 Smith J, Keating L, Flowerdew L, *et al.* An emergency medicine research priority setting partnership to establish the top 10 research priorities in emergency medicine. *Emerg Med J* 2017;**34**:454–6. doi:10.1136/emermed-2017-206702

30 Addicott R, Maguire D, Honeyman M, *et al.* Workforce planning in the NHS. London, UK: The King's Fund. 2015. https://www.kingsfund.org.uk/sites/default/files/field/field_publication_file/Workforce-planning-NHS-Kings-Fund-Apr-15.pdf (accessed 6 Feb 2019).

31 College of Emergency Medicine. Emergency medicine consultants workforce recommendations. London, UK: The College of Emergency Medicine. 2010. https://www.rcem.ac.uk/docs/Workforce/CEM5324-Emergency-Medicine-Consultants---CEM-Workforce-Recommendations-Apr-2010.pdf (accessed 6 Feb 2019).

32 Heyworth J, Clancy M, Miles G. Emergency medicine operational handbook: the way ahead. London, UK: The College of Emergency Medicine. 2011. https://www.rcem.ac.uk/docs/Policy/The%20Way%20Ahead_Final%20Dec%202011.pdf (accessed 6 Feb 2019).

FUNDED BY NIHR National Institute for Health Research

33 Reynard K, Brown R. A clinical analysis of the emergency medicine workforce crisis. *Br J Hosp Med* 2014;**75**:612–6. doi:10.12968/hmed.2014.75.11.612

34 Thornton RJ, Skalnik P. *The early writings of Bronislaw Malinowski*. Cambridge, UK: Cambridge University Press. 2006.

35 Becker HS. *Boys in white: student culture in medical school*. Piscataway, NJ: Transaction Publishers. 2002. https://www.goodreads.com/work/best_book/863100-boys-in-white

36 Liberati EG, Tarrant C, Willars J, *et al.* How to be a very safe maternity unit: an ethnographic study. *Social Science & Medicine* 2019;**223**:64–72. doi:10.1016/j.socscimed.2019.01.035

37 LeCompte MD, Schensul JJ. *Designing & conducting ethnographic research: an introduction*. Lanham, MD: Rowman Altamira. 2010.

38 Darbyshire D, Brewster L, Isba R, *et al.* Retention of doctors in emergency medicine: a scoping review protocol. *JBI Evidence Synthesis* 2020;**18**:154–162.

39 Sanday PR. The ethnographic paradigm(s). *Administrative Science Quarterly* 1979;**24**:527–38. doi:10.2307/2392359

40 Atkinson P. *Thinking ethnographically*. London, UK: SAGE. 2017.

41 Grint K. *The sociology of work*. Fourth edition. Cambridge, UK: Polity Press. 2016.

42 Edgell S, Gottfried H, Granter E, *et al. The SAGE handbook of the sociology of work and employment*. 2016.

43 Hochschild AR. *The managed heart: commercialization of human feeling*. Berkerley, CA: University of California Press. 2012. http://ebookcentral.proquest.com/lib/lancaster/detail.action?docID=870020 (accessed 25 Jun 2019).

44 Woolcock M, Narayan D. Social capital: implications for development theory, research, and policy. *World Bank Res Obs* 2000;**15**:225–49. doi:10.1093/wbro/15.2.225

45 Slattery M. *Key ideas in sociology*. Oxford, UK: Nelson Thornes. 2003.

46 Wacquant L, Akçaoğlu A. Practice and symbolic power in Bourdieu: the view from Berkeley. *Journal of Classical Sociology* 2017;**17**:55–69. doi:10.1177/1468795X16682145

47 Jafar AJN. What is positionality and should it be expressed in quantitative studies? *Emerg Med J* 2018;**35**:323–4. doi:10.1136/emermed-2017-207158

48 LeCompte MD, Goetz JP. Problems of reliability and validity in ethnographic research. *Rev Educ Res* 1982;**52**:31–60. doi:10.3102/00346543052001031

49 Rodgers BL, Cowles KV. The qualitative research audit trail: a complex collection of documentation. *Res Nurs Health* 1993;**16**:219–226.

FUNDED BY NIHR National Institute for Health Research

For peer review only - http://bmjopen.bmj.com/site/about/guidelines.xhtml

50 Varpio L, Ajjawi R, Monrouxe LV, *et al.* Shedding the cobra effect: problematising thematic emergence, triangulation, saturation and member checking. *Med Educ* 2017;**51**:40–50. doi:10.1111/medu.13124

51 Given L, Saumure K. Trustworthiness. In: *The SAGE encyclopedia of qualitative research methods*. Thousand Oaks, CA: SAGE Publications. 2008. doi:10.4135/9781412963909.n470

52 O'Reilly K. *Key concepts in ethnography*. Thousand Oaks, CA: SAGE. 2008.

53 Pink S, Morgan J. Short-term ethnography: intense routes to knowing. *Symbolic Interaction* 2013;**36**:351–61. doi:10.1002/symb.66

54 Fetterman DM. *Ethnography: step by step*. 2nd ed. Thousand Oaks, CA: Sage Publications, Inc. 1998.

55 Kvale S, Brinkmann S. *InterViews*. 3rd ed. London, UK: SAGE Publications. 2014.

56 Silverman D. *Interpreting qualitative data*. 5th ed. London, UK: SAGE. 2015.

57 QRS. *nVivo 12 Mac*. QRS International. 2018.

58 NHS Data Dictionary. Accident and emergency department type. NHS Data Model and Dictionary Version 3. 2018.https://www.datadictionary.nhs.uk/data_dictionary/attributes/a/acc/accident_and_emergency_department_type_de.asp (accessed 5 Feb 2019).

59 Idelji-Tehrani S, Al-Jawad M. Exploring gendered leadership stereotypes in a shared leadership model in healthcare: a case study. *Medical Humanities* 2018;**45**:388–98. doi:10.1136/medhum-2018-011517

60 Brummell SP, Seymour J, Higginbottom G. Cardiopulmonary resuscitation decisions in the emergency department: An ethnography of tacit knowledge in practice. *Social Science & Medicine* 2016;**156**:47–54. doi:10.1016/j.socscimed.2016.03.022

61 Murphy E, Dingwall R. Informed consent, anticipatory regulation and ethnographic practice. *Social Science & Medicine* 2007;**65**:2223–34. doi:10.1016/j.socscimed.2007.08.008

62 Braun V, Clarke V. Reflecting on reflexive thematic analysis. *Qualitative Research in Sport, Exercise and Health* 2019;**11**:589–97. doi:10.1080/2159676X.2019.1628806

63 Clarke V, Braun V. Thematic analysis. In: Lyons E, Coyle A, eds. *Analysing qualitative data in psychology*. London, UK: SAGE. 2016.

64 Braun V, Clarke V, Hayfield N, *et al.* Thematic analysis. In: Liamputtong P, ed. *Handbook of research methods in health social sciences*. Singapore: Springer Singapore 2019. 843–60. doi:10.1007/978-981-10-5251-4_103

FUNDED BY NIHR National Institute for Health Research

For peer review only - http://bmjopen.bmj.com/site/about/guidelines.xhtml

65 Terry G. Doing Thematic Analysis. In: Lyons E, Coyle A, eds. *Analysing Qualitative Data in Psychology*. Thousand Oaks, CA: SAGE. 2016.

66 Rodgers BL. Audit trail. In: *The SAGE encyclopedia of qualitative research methods*. Thousand Oaks, CA: SAGE Publications, Inc. 2008. doi:10.4135/9781412963909.n25

67 Hobbs D. Ethnography. In: Jupp V, ed. *The SAGE Dictionary of Social Research Methods*. London, UK: SAGE Publications Ltd. 2006. https://methods.sagepub.com/reference/the-sage-dictionary-of-social-research-methods (accessed 18 Oct 2019).

68 Di Domenico M, Phillips N. Participant observation. In: *Encyclopedia of case study research*. Thousand Oaks, CA: SAGE Publications, Inc. 2010. doi:10.4135/9781412957397.n244

69 Rowe WE. Positionality. In: *The SAGE encyclopedia of action research*. Thousand Oaks, CA: SAGE Publications Ltd .2014. doi:10.4135/9781446294406.n277

70 May T, Perry B. *Reflexivity: the essential guide*. London, UK: SAGE. 2017.

71 Bloor M, Wood F. *Keywords in qualitative methods*. London, UK: SAGE Publications Ltd. 2006. doi:10.4135/9781849209403

For peer review only

FUNDED BY NIHR | National Institute for Health Research

The James Lind Alliance

The James Lind Alliance (JLA) is a non-profit making initiative established in 2004. It brings patients, carers and clinicians together in Priority Setting Partnerships (PSPs) to identify and prioritise the Top 10 unanswered questions or evidence uncertainties that they agree are the most important.

The aim of this is to make sure that health research funders are aware of the issues that matter most to the people who need to use the research in their everyday lives. It is part of the NIHR (National Institute for Health Research), the research funding arm of the United Kingdom's NHS (National Health Service).

Figure 1. What is the James Lind Alliance? Information taken from the JLA website. http://www.jla.nihr.ac.uk/about-the-james-lind-alliance/ (accessed 15th January 2020)

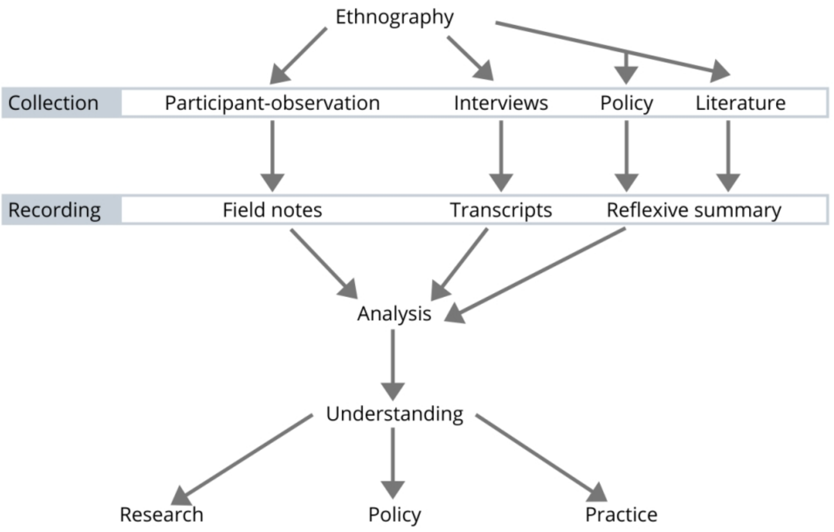

Figure 2. Summary of the ethnography. The arrows suggest a linearity to the study which is not reflected in the reality of ethnographic practice, instead the image is aiming to convey the different workstreams that come together in the analysis. The figure also fails to include the messy connections between the different parts of the study and the overlap between the intended outputs.

Stage	Description	Applied to this study
1	**Familiarisation** – involves becoming immersed in the data, getting to know it. It is about noticing things that may (or may not) be important.	This will involve reading field notes, transcripts, and other documents, and making notes about connections, possibilities, and quirks.
2	**Generating codes** – attaching labels known as codes to portions of data to create meaning at the intersection between the data set and interpretative resource that is the researcher.	Using qualitative data analysis computer software (NVivo 12 for Mac) to attach codes to data. Coding choices will be documented and attached to each code.
3	**Generating initial themes** – a good theme both identifies the area of data and tells the researcher something about it. These themes will evolve or be discarded. Sometimes called candidate themes.	Stepping back form the fine detail of the data to try and group codes that inform a specific issue.
4	**Revising/reviewing themes** – involves reviewing themes against the coded data and the entire data set. Mismatches lead to revisions.	Compiling all data for each candidate theme and reviewing against the coded data and data set in turn.
5	**Defining and naming themes** – creatively attaching names to themes that signal the core of theme. This aims to move analysis to the conceptual level while maintaining links to the data.	Thinking about the audience and research objectively to name themes that can help effectively communicate the findings.
**6**	**Writing** – producing the output from the research. The writing tests the themes. How well do they work together and in isolation? How do they stand up to the literature? These challenges lead to returning to earlier analytical stages.	Writing up the doctoral thesis, papers for medical and sociological audiences, and producing summaries for policy makers and the public.

Figure 1. The six stages of thematic analysis as applied to this study. An iterative process, it is expected that the analysis will go back and forth between stages a number of times.[63,66]

For peer review only

Lancaster University

Interview Guide

Where have all the doctors gone? An ethnographic study to explore the critical retention problem in Emergency Medicine

This guide will be used as a structure for interviews. It will allow the interviewer to draw out value from an interview and a structure to return to if the interview is going off topic. It will be shaped in response to specific observations in the fieldwork and tailored to the individual, with a focus on the overall research question: what makes doctors stay working in emergency medicine?

Pre-interview Checks

Ensure you have completed the consent procedure as outlined in the protocol.

Thank the participant for contributing.

Check the voice recording equipment is working.

Opening Question

To start the interview of ask some background questions, these will vary depending on your pre-existing knowledge of the participant.

- What is your role?
- How long have you worked in emergency medicine?
- What attracted you to work in emergency medicine?

This may direct the interview into retention, if not move onto directly asking about it.

What keeps you working in emergency medicine?

This is a very open question, follow up questions will depend upon the trajectory of the interview but may include:

Can you talk about a specific experience?
Prompts: what affected your decision? What happened after that experience?

Question Relating to Culture

How would you describe the culture of this emergency medicine department?
Is the culture here different to other EDs you have worked in?

For peer review only - http://bmjopen.bmj.com/site/about/guidelines.xhtml

Do you think that workplace culture affects staff retention?
How has it affected your work in emergency medicine?

Again, an open question, while generalities about retention and culture are interesting it is important as an interviewer to try and get to the personal experience.

Question Relating to Environment

How would you describe the work environment of this emergency medicine department?
Is the workplace here different to other EDs you have worked in?

How does the work environment affect your decision to work in EM?

Again, open questions, while generalities about retention and work environment are interesting it is important as an interviewer to try and get to the personal experience.

Digging for Depth

Having opened the conversation and grounded it in the interviewee's lived experience related to retention, the interviewer now needs to try and expand and develop any idea. Three tools are available for this:

1. *Being sensitive to cues from the previous questions and returning to them.*
2. *Asking about colleagues' experiences, building on specifics, and then returning to personal views about this.*
3. *Using a social media post to generate further discussion.*

This list is not exhaustive, nor it is a checklist. Some interviews may use all, some none, they are simply options that a skilled and attentive interviewer can apply to try and gain as much value from the interaction.

Post Interview Checklist

On completion of the interview a number of things need to be completed:

- Ensure the interviewee is alright, it is possible that the interview covered some difficult ground, the participant information sheet has sources of support.
- Thank participant for contributing.
- Are you ok? Remember the sources of support are available to the interviewer as well.
- Interviewees can be given a certificate of participation to include in their portfolio, taking part in research as a participant can help clinicians understand the process. This also acts as a formal thank you to participants.

Once the interviewee has vacated the interview space the interviewer should complete field notes related to the interview, the closer to the interview the better.

For peer review only

For peer review only - http://bmjopen.bmj.com/site/about/guidelines.xhtml

No.	Topic	Explanation and location
	**Title and abstract**	
S1	Title - Concise description of the nature and topic of the study Identifying the study as qualitative or indicating the approach or data collection methods	Title clearly states ethnographic approach, that this article is a study protocol and the problem of focus – retention of doctors in emergency medicine. Lines 4-5.
S2	Abstract - Summary of key elements of the study using the abstract format of the intended publication	Structured abstract appropriate to a protocol. Lines 22-48.
	**Introduction**	
S3	Problem formulation - Description and significance of the problem/phenomenon studied; review of relevant theory and empirical work; problem statement	Background and importance of problem outlined (lines 66-135), gap in the literature highlighted (lines 137-143).
S4	Purpose or research question - Purpose of the study and specific objectives or questions	Objectives and research questions clearly stated. Lines 156-163.
	**Methods**	
S5	Qualitative approach and research paradigm - Qualitative approach and guiding theory if appropriate; identifying the research paradigm is also recommended; rationale**	Ethnographic approach and possible theories outlined. Ethnographic paradigm highlighted. Lines 202-211.
S6	Researcher characteristics and reflexivity - Researchers' characteristics that may influence the research; potential or actual interaction between researchers' characteristics and the research	Highlighted as important in the protocol, will be detailed in the results. Lines 213-215.
S7	Context - Setting/site and salient contextual factors; rationale**	Site selection and rationale detailed. Lines 304-308.
S8	Sampling strategy - How and why research participants, documents, or events were selected; criteria for deciding when no further sampling was necessary (e.g., sampling saturation); rationale**	Sampling stratify for participant-observation and interview components detailed (lines 310-321). Discussion of theoretic saturation (lines 287-290).
S9	Ethical issues pertaining to human subjects - Documentation of approval by ethics review board and participant consent; other confidentiality and data security issues	Ethics approval documented (lines 417-419). Process of consent and capacity detailed (lines 323-382).
S10	Data collection methods - Types of data collected; details of data collection procedures including (as appropriate)	Plan to collect field notes (lines 262-263) and interview transcripts (lines 293-294) documented. Dates will be

For peer review only

	start and stop dates of data collection and analysis, iterative process, triangulation of sources/methods, and modification of procedures in response to evolving study findings; rationale**	included in report but are not stated in the protocol. Ethnographic studies are expected to evolve, how this occurs will be reported in the results but is not predicted in the protocol.
S11	Data collection instruments and technologies - Description of instruments and devices planned for data collection	Interview guides included as appendices. Use of voice recording software planned but not specified.
S12	Units of study - Number and relevant characteristics of participants, documents, or events included in the study; level of participation	Planned recruitment strategy outlined. Lines 284-286.
S13	Data processing - Methods for processing data prior to and during analysis, including transcription, data entry, data management and security, verification of data integrity, data coding, and anonymization/de-identification of excerpts	Recording and processing of field notes delineated. Interviews recorded and transcribed. Data stored and managed with nVivo 12 for Mac. Lines 293-295.
S14	Data analysis - Process by which inferences, themes, etc., were identified and developed, including the researchers involved in data analysis; usually references a specific paradigm or approach; rationale**	Data analysis plan, using reflexive thematic analysis, outlined. Lines 386-397.
S15	Techniques to enhance trustworthiness	Audit trail (lines 217-221), reflexivity and transferability discussed (lines 222-224).
	**Results/findings**	
S16	Synthesis and interpretation	Not predetermined in protocol.
S17	Links to empirical data	Not predetermined in protocol.
	**Discussions**	
S18	Integration with prior work, implications, transferability, and contribution(s) to the	Not predetermined in protocol.
S19	Limitations - Trustworthiness and limitations of findings	Anticipated limitations discussed. Lines 404-413.
	**Other**	
S20	Conflicts of interest - Potential sources of influence or perceived influence on study conduct and conclusions; how these were managed	Lines 459-461.
S21	Funding - Sources of funding and other support; role of funders in data collection, interpretation, and reporting	Lines 465-467.

For peer review only

Table 1. SRQR Table - Standards for Reporting Qualitative Research. The SRQR tool is designed for reporting of completed studies, as such certain aspects are merely acknowledged are requiring inclusion in the final report. The table allows the reader to map the study protocol to a recognised quality indicator. The topic descriptions have been maintained, but edited for brevity, the full description can be found in the original publication.[1]

For peer review only

BMJ Open

'Where have all the doctors gone?' A protocol for an ethnographic study of the retention problem in Emergency Medicine

Journal:	*BMJ Open*
Manuscript ID	bmjopen-2020-038229.R2
Article Type:	Protocol
Date Submitted by the Author:	26-Oct-2020
Complete List of Authors:	Darbyshire, Daniel; Lancaster University Faculty of Health and Medicine, Medical School; Salford Royal NHS Foundation Trust, Emergency Department<br>Brewster, Liz; Lancaster University, Lancaster Medical School<br>Isba, Rachel; Lancaster University Faculty of Health and Medicine, Medical School; North Manchester General Hospital, Children's Accident and Emergency Department<br>Body, Richard; The University of Manchester, Division of Cardiovascular Sciences; Manchester University NHS Foundation Trust, Emergency Department<br>Goodwin, Dawn; Lancaster University Faculty of Health and Medicine, Medical School
**Primary Subject Heading**:	Emergency medicine
Secondary Subject Heading:	Health services research, Health policy, Medical education and training, Qualitative research, Sociology
Keywords:	ACCIDENT & EMERGENCY MEDICINE, Health policy < HEALTH SERVICES ADMINISTRATION & MANAGEMENT, MEDICAL EDUCATION & TRAINING, QUALITATIVE RESEARCH

SCHOLARONE™
Manuscripts

I, the Submitting Author has the right to grant and does grant on behalf of all authors of the Work (as defined in the below author licence), an exclusive licence and/or a non-exclusive licence for contributions from authors who are: i) UK Crown employees; ii) where BMJ has agreed a CC-BY licence shall apply, and/or iii) in accordance with the terms applicable for US Federal Government officers or employees acting as part of their official duties; on a worldwide, perpetual, irrevocable, royalty-free basis to BMJ Publishing Group Ltd ("BMJ") its licensees and where the relevant Journal is co-owned by BMJ to the co-owners of the Journal, to publish the Work in this journal and any other BMJ products and to exploit all rights, as set out in our licence.

The Submitting Author accepts and understands that any supply made under these terms is made by BMJ to the Submitting Author unless you are acting as an employee on behalf of your employer or a postgraduate student of an affiliated institution which is paying any applicable article publishing charge ("APC") for Open Access articles. Where the Submitting Author wishes to make the Work available on an Open Access basis (and intends to pay the relevant APC), the terms of reuse of such Open Access shall be governed by a Creative Commons licence – details of these licences and which Creative Commons licence will apply to this Work are set out in our licence referred to above.

Other than as permitted in any relevant BMJ Author's Self Archiving Policies, I confirm this Work has not been accepted for publication elsewhere, is not being considered for publication elsewhere and does not duplicate material already published. I confirm all authors consent to publication of this Work and authorise the granting of this licence.

For peer review only

Title Page

Title

'Where have all the doctors gone?' A protocol for an ethnographic study of the retention problem in Emergency Medicine

Corresponding author: Dr Daniel Darbyshire, B32 Furness College, Lancaster University, Bailrigg, LA1 4YG. d.darbyshire@lancaster.ac.uk (+44)0 7757119141

Authors and affiliations

Daniel Darbyshire (1,2), Liz Brewster (1), Rachel Isba (1,3), Richard Body (4,5), Dawn Goodwin (1)

(1) Lancaster Medical School, Bailrigg, UK. (2) Emergency Department, Salford Royal Hospital NHS Foundation Trust, Salford, UK. (3) Children's Accident and Emergency Department, North Manchester General Hospital, Manchester, UK. (4) Division of Cardiovascular Sciences, The University of Manchester, Manchester, UK. (5) Emergency Department, Manchester University NHS Foundation Trust, Manchester, UK

Word count = 3999

Keywords - Ethnography, staffing, emergency medicine, sustainability.

Abstract

Introduction

'Emergency medicine (EM) in the United Kingdom (UK) has a medical staffing crisis.' Inadequate staffing, in EM and across healthcare, is a problem that affects the quality of patient care globally. Retention of doctors in EM is a particularly acute problem in the UK's National Health Service (NHS). Sustainable careers in healthcare are gaining increasing attention at a national and international policy level, but research to understand the factors that facilitate retention is lacking.

This study aims to develop understanding of what drives retention of doctors in EM by focusing on those who remain in these careers, where previous research has targeted those who have left. By addressing the problem of retention in a different way, using innovative methods in this context, we aim to develop a deeper and more nuanced understanding of sustainable careers in EM.

Methods and analysis

This is an ethnographic study combining participant observation in two emergency departments, interviews with doctors from these departments, from organisations with influence or interest at a policy level and with doctors who have left emergency medicine. The analyses will integrate detailed workplace observation alongside key academic and policy documents using reflexive thematic analysis.

Ethics and dissemination.

Approvals have been obtained from Lancaster University via the Faculty of Health and Medicine Research Ethics Committee (FHMREC18058) and the Health Research Authority (IRAS number 256306). The findings will inform understanding of sustainable careers in EM

FUNDED BY NIHR National Institute for Health Research

For peer review only - http://bmjopen.bmj.com/site/about/guidelines.xhtml

that may be transferable to other settings, professions, and locations that share key characteristics with EM such as paediatrics, emergency nursing, and general practice. Findings will be disseminated through a series of academic publications and presentations, through local and specialty research engagement, and through targeted policy statements.

Strengths and Weaknesses

- Focuses on a national research priority and a global problem.
- Takes a novel approach to understanding the retention problem.
- Engaged with policy makers and the public throughout the research.
- Some aspects of retention will vary by context.
- Policies and practice will evolve during the research, making it harder to unpick some of the findings.

For peer review only

FUNDED BY NIHR | National Institute for Health Research

Main Text

Main text separated under appropriate headings and subheadings using the following hierarchy: BOLD CAPS, bold lower case, Plain text, Italics

INTRODUCTION

The specialty of emergency medicine (EM) provides care across the spectrum of illness and injury to people of all ages. It operates all day, every day and in the United Kingdom (UK) has seen demand increase year on year, with declining performance in terms of patients having their emergency department (ED) visit concluded within four hours, and a rise in numbers of patients spending greater than 12 hours in the ED.[1] Much of this declining performance is attributed to factors outside of the ED, with exit block due to insufficient hospital beds and community services being the most frequently cited contributing factors.[2,3] Arguably, the most important factor for high-quality care in the ED that departments have some control over, is staffing.

Unfortunately, 'Emergency Medicine in the UK has a medical staffing crisis.'[4] Starting with the formation of the Emergency Medicine Taskforce, set up in 2011,[4] the specialty has sought to change practice, policy and culture though innovations such as the 'less than full time training' pilot,[5] increasing recognition of the particular stresses a career in EM brings and a focus on sustainable careers.[6,7] This has led to EM growing rapidly in terms of both consultant and trainee numbers but demand for care has outstripped staffing growth and looks to continue to do so.[8]

The EM workforce problem is occurring in the context of staffing shortages in much of the United Kingdom's National Health Service (NHS)[9] and on a background of demand outstripping growth in the global healthcare workforce.[10] The picture is complex. The NHS employs around one million people, with the workforce increasing by around 1.8% from July 2017 to 2018. However, organisations report collective vacancies across the NHS in the region of 100,000 and falling numbers of key staff groups.[9] The *NHS Long Term Plan*,[11] published in January 2019 (updated August 2019), followed by the *Interim People Plan*,[12] outlined the UK Government's proposed solutions for staffing the NHS. The plans – which focused on staff development, empowerment, and improving leadership culture – were broadly welcomed.[13] There were, however, concerns, for example about the time lag between increasing training numbers and generating qualified practitioners in employment, with potential over-reliance on international recruitment in the interim.[13,14]

From recruitment to retention

EM historically had a recruitment problem, but after much work by the then College of Emergency Medicine (now the Royal College of Emergency Medicine) and Health Education England, amongst others, in improving the image of the specialty and the experience of trainees, fill rates for EM training posts reached 96% in 2011.[15] Recruitment has since fallen from the 2011 high, yet it remains strong and competitive, when compared with other specialities, at 86% in 2019 (compared to 77% for internal medicine and 69% for paediatrics).[16] However, attrition from training programmes is unsustainably high, with The King's Fund (an independent English think tank) reporting that: 'problems with retention mean it [emergency medicine] has the greatest attrition rate of any medical

FUNDED BY NIHR National Institute for Health Research

specialty, with almost 50 per cent of registrar doctors in their third or fourth year of training resigning.'[17]

Following on from a 2014 survey of trainees that looked at the attractiveness of a career in EM,[18] the Emergency Medicine Trainees' Association now conduct its own annual surveys.[19–21] These surveys elucidate the challenges faced by trainees, specifically burnout, poor working conditions, and workload pressures. This is supported by the General Medical Council's national training survey which showed EM as the specialty with the highest work intensity[22] and by the high attrition from the emergency medicine specialty training programme.[23] The data on trainees is reflected for consultants and staff and associate specialist grades, with all groups being undersubscribed and experiencing exodus.[23]

Recruiting and retaining EM clinicians is fundamentally connected to the provision of high-quality clinical care. More experienced and senior doctors deliver better care, performing fewer unnecessary tests and getting fewer complaints, and experience is only gained by retaining doctors within the specialty.[24–26] Retention of doctors affects wider health and care services in two main ways. Firstly, the presence of doctors with more experience results in efficiency savings both in the ED and downstream in a patient's hospital journey, with recent studies finding a greater than 20% reduction in admission rates, reduced waiting times, and cost savings of £3 million to one hospital.[24,25] The introduction of the Major Trauma Networks in 2010 with consultant-led care of severely injured patients in London saved an estimated 58 lives in the first year.[26] Secondly, staff turnover is costly and disruptive. When ED staff leave it is highly unusual for replacements to be found quickly, if at all, often leading to locum (temporary) doctors filling in who are 'generally less efficient, and less safe, than permanent members of staff'.[27] Costs in the UK are not clear but an approximate figure for replacing one fully trained emergency physician in the US is $164,000.[28]

We would argue that the evidence is therefore clear that staff retention is important for quality emergency care. What is not clear is what drives retention and what allows people to have sustainable careers. This knowledge gap was also identified in the 2017 James Lind Alliance (see figure 1) priority setting partnership for EM research. Number four in their list of the top ten research priorities in EM asked: 'with regard to how ED staff development is managed, what initiatives can improve staff engagement, resilience, retention, satisfaction, individuality and responsibility?'[29] This priority was supported by calls from various other stakeholders to address the issue of retention including The King's Fund,[17,30] Health Education England[15], and the Royal College of Emergency Medicine.[27, 31–33]

The proposed study aims to address retention of doctors in EM – an innovative approach, contrasting with previous work that has focused predominantly on why people leave. The ethnographic methodology is also innovative in this setting as the retention problem has not previously been explored using this approach. By developing fresh insights into the complex and interacting factors that positively influence retention, we hope to inform future efforts aimed at reducing exodus from the specialty, at both a research and policy level.

Research aim and objectives

For peer review only - http://bmjopen.bmj.com/site/about/guidelines.xhtml

The aim of this study is to gain a deep understanding of retention in EM, in order to elucidate how retention is made possible. This aim requires a study design that can tackle complexity and develop new understandings. The objectives are to:

- understand in detail the day-to-day lived experience of EM doctors, to identify and explore factors influencing retention;
- situate these descriptions within the current educational and health policy contexts;
- advance the debate and make policy and practice recommendations based on a detailed understanding of retention of doctors in EM.

METHODS

Overview

We will conduct an ethnographic study of EM doctors in two type 1 EDs in the North West of England. Ethnography is derived from anthropology and social sciences and is historically associated with gaining cultural understandings about groups, with the origins of the practice going back to colonial-era studies of 'exotic' civilisations.[34] Its use within health research is broad and well-established, from a 1961 study of medical student culture[35] through to a recent study trying to understand why one particular maternity unit performs highly.[36]

Ethnography is 'a systematic approach to learning about social and cultural life of communities, institutions, and other settings'.[37] Key features of ethnography relevant to this study are that it is investigative, the primary tool of data collection is the researcher, and that it 'emphasises and builds on the perspective of the people in the research setting'.[37]

Ethnography creates understanding through description and analysis. This usually involves going to a place and collecting data – which is often a detailed description of what happens in that place along with the accompanying talk – over a period of time. What information is recorded, how long the researcher spends there, and what other forms of data are collected, depends on the context of the research and question being asked. For this research, ethnography refers to the totality of the study including the time spent observing in the department (referred to as participant-observation hereafter), interviews, and policy documents.

This ethnographic study will involve the lead researcher spending time in two different EDs, trying to understand what allows doctors to have sustainable careers. This will involve observing the people, space, and happenings in the departments, and speaking to those in these spaces, keeping the focus on retention. Without aiming to predict retention-related behaviours, this may mean observing humour, conversations between members of staff, or how they work within the challenging working environments. This will be supported by interviews with doctors (conducted away from the department) and with others who can inform the research aim and objectives, and by critical review of policy and academic literature relating to retention. The literature search strategy, in the form of a scoping review, is described elsewhere.[38] Each step in the process is expanded on below and summarised in figure 2. The study is inherently iterative, observations and interviews will

FUNDED BY NIHR National Institute for Health Research

For peer review only - http://bmjopen.bmj.com/site/about/guidelines.xhtml

inform subsequent observations and interviews, and the study plan may adapt to analysis, and opportunities and challenges in the field.

This study subscribes to the ‘ethnographic paradigm’[39], by this we mean that we don’t see value in predetermining a particular way of seeing the world, and agree with Atkinson when he writes that ‘ideas can and should come from a variety of sources’.[40] This study draws on the sociological practice of ethnography and as such the findings will be analysed, interpreted, and challenged through a number of sociological theories. These will include, but are not limited to, theories of work[41,42] such emotional labour[43] and social capital,[44] theories of community such as Tönnies discussion of Gemeinschaft and Gesellschaft (community and society)[45], and Bourdieu’s conception of symbolic power.[46] This list is not intended to be prescriptive – it aims to show theories that may prove informative.

As the study is built on the interpretation of DD (the researcher collecting the data and performing the analysis), a discussion of their positionality will be included when the study reports.[47] This is an integral part of reflexive thematic analysis described below, ‘addressing threats to credibility in ethnography requires different techniques from those used in experimental studies.’[48] Our study aims to produce a clear audit trail, of which publishing this protocol is a part.[38,49] Other important parts of the audit trail are notes made during observations about why the researcher has chosen to observe at particular times and locations, documents generated around meetings of the research team, and documents from peer-review, ethics, and the funder which have shaped the study.[49] We are aiming for transferability not generalisability, reflecting the importance of context,[50] and will utilise reflexive thematic analysis, sense checking from the public involvement group. We aim for confirmability by aligning interpretations and data in the final report.[51]

Patient and public involvement

The public has been involved in the development of this study at several levels and at different key points in the development of the protocol. The aforementioned James Lind Alliance priority setting exercise demonstrated the public’s belief that the topic areas is important.[29] To confirm this, with the support of the NIHR Research Design Service North West and the Research and Development Department at Blackpool Victoria Hospital, we ran a public involvement event. During this event, members of the public agreed with the James Lind Alliance that exploring factors related to the retention of doctors in EM merited investigation. Participants thought the proposed methods were acceptable to patients, but questioned whether ethnography would be seen as acceptable to a clinical audience. A member of the initial patient involvement group has volunteered to be further involved in the study by becoming a member of the steering committee and by reviewing the conclusions we gather during the scoping review part of the study.[38]

As the main focus of the study is EM doctors, we also sought their views as part of our engagement strategy. We presented the research plan, along with some of the preliminary findings from the scoping review,[38] at the Emergency Medicine Trainees’ Association National Conference in Cardiff, in November 2018. Feedback was obtained during the question and answer question and by providing a link to an online form. This process was repeated at the Oxford Society for Emergency Medicine Conference and the Association for the Study of

FUNDED BY NIHR | National Institute for Health Research

For peer review only

Medical Education Researching Medical Education Meeting in London, both in December 2018. Thirteen attendees provided written feedback, including one offer to help, which led to recruitment of a second reviewer for the scoping review, with many more providing verbal comments and questions. Including interviews with doctors who have left was suggested during this professional engagement, and again during peer review from the NIHR when funding was being obtained, and subsequently included in the research plan.

Participant-observation

Participant-observation refers to the time spent in the research setting watching, listening, and documenting. It is strongly linked with ethnography and is sometimes synonymous with field work. These terms are contentious[52] and for clarity we refer to participant-observation as the component of ethnography that occurs in the research setting. All data collection will be conducted by DD who will undertake observation at two sites, each for a period of twelve weeks. At each site they will perform observations in all parts of the department and at all times of the day. A day of participant-observation will be divided in two. The first half of the day will be spent conducting observations and writing field notes, incorporate talking with staff and documenting what they say, and the researcher's thoughts about the encounter. We will investigate the working environment, interaction between staff, and behaviours related to retention. Clinical encounters are not the focus of this study, they have been studied from multiple perspectives and are well-understood by the primary audience for this research–emergency physicians and EM leaders. The field notes will be handwritten in a notebook. The other half of the day will involve typing up, reflecting on, and expanding field notes. We aim to perform four days of observation each week by scheduling forty-eight days over the twelve-week period with start times reflecting the shift patterns locally, but to include days, evening, nights and weekends. A total of twenty-four weeks' participant-observation was chosen as it will allow DD to participate in observations at all times, with flexibility to repeat observations at key times or with specific key informants. Traditionally ethnographic observation has been conducted over a much greater period of time than the one that proposed, to allow for a period of familiarisation. This process will be accelerated in this study due to DD's pre-existing knowledge of EM and EDs (as a senior trainee in EM). This more focused form of ethnography has a developing literature for its use in complex healthcare settings[53] and based on this work, and the intensity of the observation within the twelve-week period, it is estimated that this will be sufficient to meet the aims of the study.

Interviews

In addition to the participant-observation conversations described above, we will conduct interviews, reserving the term for the physically separate, planned, audio-recorded and transcribed encounters. DD will conduct all the interviews, aiming to complete around 40. Ten interviews with doctors across all levels of seniority from each of the two research sites, ten interviews with individuals within key organisations relevant to retention in EM (such as the Royal College of Emergency Medicine, the Emergency Medicine Trainees' Association, Health Education England, and NHS Improvement), and ten with doctors who have left EM. Potential participants for interviews will be identified during the periods of participant-observation and the literature review and supplemented by recommendations obtained from interviewees. Interviewing ten doctors from each research site would represent between a quarter and a third of doctors at each level present on a rota at any time. The sample size has been agreed

FUNDED BY NIHR | National Institute for Health Research

upon by discussion within the research team, who have extensive experience with this methodology, and by consulting key texts, and represents an estimation for when theoretical saturation (see glossary) might be achieved.[54–56]

Interviews will take place in a neutral space which allows for private conversation. The interview guide is available in Appendix 1. However, it is expected to evolve as the study progresses, based on pilot interviews, and ongoing analysis. Interviews will be digitally recorded, transcribed, and imported into nVivo 12,[57] the qualitative data analysis computer package that facilitates the organisation and analysis of research data. After each interview DD will record reflections in the field notes.

Policy and literature

Policy documents (grey literature) will be identified through the aforementioned scoping review[38] and from the other aspects of the ethnography. Key documents will be chosen based on relevance to research context and incorporated into the analysis. A similar process will be used for academic literature.

Inclusion criteria

Participant observation sites were selected based on the following factors:

- presence of foundation, core, and higher trainees;
- presence of staff and associate specialists;
- Type 1 ED (major ED, providing a consultant-led 24-hour service with full resuscitation facilities).[58]

Interview and observation candidates

Within the two EDs the focus for participant observation, and interview candidates is:

- doctor;
- working in the study site;
- staff member for at least 4 weeks
- aim to include a maximally inclusive representation of the department, including age, gender, seniority, international medical graduates, and UK trained doctors.

The inclusion criteria are intended to be broad and inclusive. The 4-week criteria were chosen to avoid interviewing very new staff members at the start of their work. The longitudinal nature of the ethnography means that these candidates may be eligible once they have been in the department for a month or more.

Consent

Participant-observation

Informed consent needs to match the research and the environment in which it is being undertaken. We are not alone in making this point, several previous studies within the NHS[36,59] and the ED[60] have taken a proportionate route, clearly demonstrating the acceptability and utility of such an approach.

To ensure participants are informed of the study protocol, aims, and data collection methods, a continuous information sharing approach will be taken, mirroring the studies referenced above. The research has already been explained to the senior leadership at each study site, along with the research and development leads. To complement this, we will

FUNDED BY NIHR | National Institute for Health Research

For peer review only - http://bmjopen.bmj.com/site/about/guidelines.xhtml

present the research plan to the medical and nursing staff at scheduled meetings such as junior doctors teaching, band 5, 6 and 7 nursing meetings, consultant meetings and departmental research and governance meetings. We will advertise the start of the study with posters giving people the opportunity to ask questions, and state objections, before it starts. Emails will be circulated to all ED staff informing them of the study. In addition, we will offer a brief explanation to staff members who the researcher interacts with in the department, as both part of the consent process and as a way to start conversations as part of the research method.

When introducing himself in the research setting DD will reiterate the voluntary nature of the study and that it is ok for them to ask for observation to stop, either temporarily or until further notice. Based on previous studies in the ED this is unlikely to occur frequently, and as such we intend, if this happens, to relocate to a different part of the department to continue observation. If the objection is not time limited then the researcher would not attend when that person was on shift, the 24-hour nature of EM makes this feasible. Should an individual ask for more information about the study we will provide them with a short summary of the study, a link to this protocol and contact details for the research team.

Patients are not participants in this study, and no patient identifiable data will be recorded, but they, and other staff members, do occupy the research space. Their confidentiality and acceptance of the research is vital. We believe it is unfeasible, overly burdensome, and unnecessary to obtain consent from patients transiting through the ED. Either DD will introduce themselves or will be introduced by the member of staff they are accompanying. If a patient does not want to have the care they receive observed, DD will withdraw from that episode of care. DD will rely on his experience and expertise of working within this environment to proportionally address this issue in real time.

Informed consent for ethnographic study is different to other types of research, particularly so in unpredictable settings such as the ED. The use of the standard written consent procedure is overly burdensome to the researcher and participant, not feasible, and would bar patients and staff members who occupy these spaces from benefiting from this type of research.[61]

Interviews

Formal written consent, supported by a participant information sheet, will be obtained for the formal, recorded, interviews. Consent will be obtained by DD prior to conducting the interview. Having expressed initial interest in participating, potential interviewees will be provided with a copy of the consent form and participant information sheet. On the day of the interview, the interviewee will be given another opportunity to review the participant information sheet and consent form, with DD on hand to answer any questions. Some interviewees, especially those with high-profile roles in national organisations, may feel that anonymisation is either impossible or not in their interests; they will be given the option of participating in the study in a non-anonymised fashion. They will retain the right to have their interview anonymised, and all interviewees have the right to withdraw, up to the point of publication.

ANALYSIS

For peer review only

During the data collection phase of the study, initial analysis will take the form of reflective analytical notes recorded in the fieldnotes, these observations and interpretations guide the ongoing analysis. Once data collection is complete, a more thorough, systematic analysis of fieldnotes, interview transcripts and literature and policy documents will be completed utilising reflexive thematic analysis.[62–64] This process leads to generation of themes – these are not groups of data that are about the same thing, but 'stories about particular patterns of shared meaning across the dataset'.[62] Reflexive thematic analysis emphasises the effortful nature of analysis, in that themes are not found within the data, rather they are created though analytical work. This highlights the role of the researcher in generating the analysis, laying this open to scrutiny. The analysis will use the six-stage approach to thematic analysis (see Figure 3 for a summary).[62,65] These stages are descriptive and supportive, as opposed to having clear divisions. It is expected that the analysis will go back and forth through the stages multiple times.

Limitations

The study is geographically limited to the North West of England. Much of the activity of EDs is similar nationally, but local differences will mean the findings will require interpretation and translation to other locales.

The study is based at two sites. There is no reliable objective way of stating if the sites have high or low retention, relative to the national picture. If they are extreme outliers it may be difficult for other sites to interpret the findings.

This study is focused solely on doctors working in EDs, there may be some transferability to other professions or working environments, but this is a limitation.

Data collection by someone without experience of the ED may lead to different conclusions, though this might be at the expense of access problems and detailed understanding of the work in the ED.[52]

ETHICS AND DISSEMINATION

Approval from Lancaster University via the Faculty of Health and Medicine Research Ethics Committee (FHMREC18058) was received on 15th April 2019. Health Research Authority approval was formalised on 11th November 2019, IRAS number 256306. Traditional academic dissemination will occur locally through seminars and lectures, and to a broader audience though conference presentations and academic publications. Dissemination to the public will be facilitated by presentations at knowledge translation events and by engaging with local media and research translation services such as The Conversation. Policy briefs summarising the findings will be provided for key audiences such as NHS England, Health Education England, and the Royal College of Emergency Medicine.

Glossary

Audit trail – a thorough collection of documentation regarding all aspects of the research. Qualitative research often evolves though the iterative process of data collection and

FUNDED BY NIHR | National Institute for Health Research

For peer review only

analysis, requiring the researcher to make frequent decisions which alter the course of the research. Documents that form this trail of evidence include field notes, a research diary, and correspondence.[49,66]

Ethnography – a method based on description and the key assumption that 'personal engagement with the subject is the key to understanding a particular culture or social setting'.[67] It can involve many different ways of collecting data, but is strongly associated with participant-observation and field work.

Participant-observation – a key tool in ethnographic investigation and in effect a combination of a wide variety of methods including observation, informal interviews and conversations, combined with analysis of other materials encountered in the research setting. The term describes the two activities that the researcher does, to a greater or lesser extent, to gain access and trust in the field site and to develop understanding of what is happening there.[68]

Positionality – how the researcher is positioned with respect to the social and political context of the study. Affects all aspects from formation of the research question to analysis. Making this explicit allows the reader to interpret the findings in context.[47,69]

Reflexivity – conscious examination and explanation of how the researcher's positionality influences and is influenced by the research as it progresses.[70]

Theoretical saturation – the continuation of sampling and data collection until no new conceptual insights are generated. At this point the researcher has provided repeated evidence for his or her conceptual categories.[71]

Trustworthiness – the ways in which a qualitative researcher ensures quality in their research, specifically in terms of transferability and credibility.[51] Moves discussions about research quality away from the quantitative paradigm of triangulation and generalisability.

Competing interests statement

This publication presents independent research funded by the National Institute for Health Research (NIHR). The views expressed are those of the author(s) and not necessarily those of the NHS, the NIHR or the Department of Health and Social Care.

All authors have completed the ICMJE uniform disclosure form at www.icmje.org/coi_disclosure.pdf and declare: DD had financial support from the NIHR, BMA Foundation and the Royal College of Emergency Medicine for the submitted work; no financial relationships with any organisations that might have an interest in the submitted work in the previous three years; no other relationships or activities that could appear to have influenced the submitted work.

Funding statement

Daniel Darbyshire is funded by a National Institute for Health Research (NIHR) Doctoral Fellowship for this research project. Initiation of the study was supported by the BMA Foundation Kathleen Harper Award and the RCEM Young Investigator Award.

FUNDED BY

NIHR | National Institute for Health Research

For peer review only

Contributorship statement

DD conceived the project. DD, LB, RB and DG developed the initial proposals for funding. DD, LB, DG, RI and RB developed the protocol. DD produced the initial draft of the manuscript. LB and DG are providing direct PhD supervision for DD. RB and RI are supporting the PhD with specialty and institutional expertise respectively. All authors contributed to and revised the final manuscript.

Acknowledgements

The authors would like to acknowledge the ongoing work of Gavin Quick and Anne Clark for their public and patient involvement role in developing the protocol.

References

1 Royal College of Emergency Medicine. RCEM Winter flow project: final report July 2019. London, UK: Royal College of Emergency Medicine. 2019. https://rcem.ac.uk/docs/RCEM_Winter_Flow_Project_Final_Report_2019.pdf (accessed 11 Oct 2019).

2 Mason S, Knowles E, Boyle A. Exit block in emergency departments: a rapid evidence review. *Emerg Med J* 2017;**34**:46–51. doi:10.1136/emermed-2015-205201

3 Knowles E, Mason SM, Smith C. Factors associated with exit block and impact on the emergency department. *Emerg Med J* 2017;**34**:61–2. doi:10.1136/emermed-2015-205202

4 Hughes G. The emergency medicine taskforce: an interim report. *Emerg Med J* 2013;**30**:348–348. doi:10.1136/emermed-2012-202260

5 Clancy M. The Less Than Full Time (LTFT(3)) working in emergency medicine pilot final report. London, UK: The Royal College of Emergency Medicine. 2019. https://www.rcem.ac.uk/docs/Training/LTFT_pilot_FINAL_REPORT-for_website.pdf (accessed 11 Oct 2019).

6 Hewitt S, Kennedy U. EM-POWER: a wellness compendium for EM. London, UK: The Royal College of Emergency Medicine. 2019. https://www.rcem.ac.uk/docs/Sustainable%20Working/0.%20Wellness%20Compendium%20(Apr%202019).pdf (accessed 11 Oct 2019).

7 RCEM Sustainable Working Practices Committee. EMPOWER: A guide to engage and retain your established EM staff. London, UK: Royal College of Emergency Medicine. 2018. https://www.rcem.ac.uk/docs/Workforce/Engage%20and%20Retain%20Sept%202018.pdf (accessed 20 Aug 2019).

8 Smith E, RCEM Service Design and Configuration Committee. RCEM workforce recommendations 2018: consultant staffing in emergency departments in the UK. London, UK: The Royal College of Emergency Medicine. 2018.

For peer review only

FUNDED BY NIHR National Institute for Health Research

For peer review only - http://bmjopen.bmj.com/site/about/guidelines.xhtml

https://www.rcem.ac.uk/docs/Workforce/RCEM%20Workforce%20Recommendations%202018%20-%20Consultant%20Staffing.pdf (accessed 6 Feb 2019).

9 Buchan J, Charlesworth A, Gershlick B, *et al.* A critical moment: NHS staffing trends, retention and attrition. London, UK: The Health Foundation. 2019. https://www.health.org.uk/sites/default/files/upload/publications/2019/A%20Critical%20Moment_1.pdf (accessed 11 Oct 2019).

10 Britnell M. Human: Solving the global workforce crisis in health care. The Nuffield Trust. 2019. https://www.nuffieldtrust.org.uk/news-item/human-solving-the-global-workforce-crisis-in-health-care (accessed 10 Jun 2019).

11 NHS England. The NHS long term plan. London, UK: NHS. 2019. https://www.longtermplan.nhs.uk/wp-content/uploads/2019/08/nhs-long-term-plan-version-1.2.pdf (accessed 11 Oct 2019).

12 NHS Improvement. Interim NHS people plan. London, UK: NHS Improvement. 2019. https://improvement.nhs.uk/resources/interim-nhs-people-plan/ (accessed 10 Jun 2019).

13 Bailey S. The King's Fund responds to the interim NHS people plan. London, UK: The King's Fund. 2019. https://www.kingsfund.org.uk/press/press-releases/interim-nhs-people-plan (accessed 11 Oct 2019).

14 Campbell J, Dussault G, Buchan J, *et al.* A universal truth: no health without a workforce. Geneva, Switzerland: Global Health Workforce Alliance and World Health Organization. 2013. https://www.who.int/workforcealliance/knowledge/resources/GHWA-a_universal_truth_report.pdf (accessed 10 Jun 2019).

15 Health Education England. Emergency medicine: background to HEE proposals to address workforce shortages. London, UK: Health Education England. 2013. https://www.hee.nhs.uk/our-work/emergency-medicine (accessed 6 Feb 2019).

16 Health Education England. Specialty recruitment: round 1 - acceptance and fill rate. London, UK: Health Education England. 2018.https://www.hee.nhs.uk/our-work/medical-recruitment/specialty-recruitment-round-1-acceptance-fill-rate (accessed 9 Aug 2019).

17 The King's Fund. What's going on in A&E? London, UK: The King's Fund. 2014. https://www.kingsfund.org.uk/projects/urgent-emergency-care/urgentand- emergency-care-mythbusters (accessed 5 Feb 2019).

18 Joy T, Moran J, Hassan T, *et al.* The attractiveness of a career in Emergency Medicine: A survey of UK trainees. London, UK: The College of Emergency Medicine. 2014. http://www.emtraineesassociation.co.uk/emta-surveys.html# (accessed 5 Feb 2019).

19 Archer K, Bailey J, Jenkinson E. EMTA trainee survey 2015. London, UK: Emergency Medicine Trainees Association. 2016. http://www.emtraineesassociation.co.uk/emta-surveys.html# (accessed 4 Feb 2019).

FUNDED BY
NIHR | National Institute for Health Research

20 Bailey J, Archer K, Stewart P, *et al.* EMTA survey 2016. London, UK: Emergency Medicine Trainees Association 2017. http://www.emtraineesassociation.co.uk/emta-surveys.html# (accessed 4 Feb 2019).

21 Bailey J, Mashru A, Stewart P, *et al.* EMTA survey 2017. London, UK: Emergency Medicine Trainees Association. 2018. http://www.emtraineesassociation.co.uk/emta-surveys.html# (accessed 4 Feb 2019).

22 General Medical Council. National training survey 2019: Initial findings report. Manchester, UK: GMC 2019. https://www.gmc-uk.org/-/media/national-training-surveys-initial-findings-report-20190705_2.pdf?la=en (accessed 11 Oct 2019).

23 Health Education England, NHS England, NHS Improvement, *et al.* Securing the future workforce for emergency department in England. London, UK: NHS Improvement. 2017. https://improvement.nhs.uk/documents/1826/Emergency_department_workforce_plan_-_111017_Final.3.pdf (accessed 28 May 2019).

24 White AL, Armstrong PAR, Thakore S. Impact of senior clinical review on patient disposition from the emergency department. *Emergency Medicine Journal* 2010;**27**:262–5. doi:10.1136/emj.2009.077842

25 Geelhoed GC, Geelhoed EA. Positive impact of increased number of emergency consultants. *Arch Dis Child* 2008;**93**:62–4. doi:10.1136/adc.2007.122531

26 Cole E, Lecky F, West A, *et al.* The impact of a pan-regional inclusive trauma system on quality of care. *Ann Surg* 2016;**264**:188–94. doi:10.1097/SLA.0000000000001393

27 Higginson I. Medical and practitioner staffing in emergency departments. London, UK: The College of Emergency Medicine. 2015. https://www.rcem.ac.uk/docs/Workforce/RCEM%20Medical%20and%20Practitioner%20Staffing%20in%20EDs.pdf (accessed 6 Feb 2019).

28 Shah S. The real cost of emergency department physician turnover. Pensacola, FL,: Studer Group. 2016. https://www.studergroup.com/resources/articles-and-industry-updates/insights/january-2016/the-real-cost-of-emergency-department-physician-tu (accessed 12 Feb 2019).

29 Smith J, Keating L, Flowerdew L, *et al.* An emergency medicine research priority setting partnership to establish the top 10 research priorities in emergency medicine. *Emerg Med J* 2017;**34**:454–6. doi:10.1136/emermed-2017-206702

30 Addicott R, Maguire D, Honeyman M, *et al.* Workforce planning in the NHS. London, UK: The King's Fund. 2015. https://www.kingsfund.org.uk/sites/default/files/field/field_publication_file/Workforce-planning-NHS-Kings-Fund-Apr-15.pdf (accessed 6 Feb 2019).

31 College of Emergency Medicine. Emergency medicine consultants workforce recommendations. London, UK: The College of Emergency Medicine. 2010.

For peer review only

FUNDED BY NIHR National Institute for Health Research

https://www.rcem.ac.uk/docs/Workforce/CEM5324-Emergency-Medicine-Consultants---CEM-Workforce-Recommendations-Apr-2010.pdf (accessed 6 Feb 2019).

32 Heyworth J, Clancy M, Miles G. Emergency medicine operational handbook: the way ahead. London, UK: The College of Emergency Medicine. 2011. https://www.rcem.ac.uk/docs/Policy/The%20Way%20Ahead_Final%20Dec%202011.pdf (accessed 6 Feb 2019).

33 Reynard K, Brown R. A clinical analysis of the emergency medicine workforce crisis. *Br J Hosp Med* 2014;**75**:612–6. doi:10.12968/hmed.2014.75.11.612

34 Thornton RJ, Skalnik P. *The early writings of Bronislaw Malinowski*. Cambridge, UK: Cambridge University Press. 2006.

35 Becker HS. *Boys in white: student culture in medical school*. Piscataway, NJ: Transaction Publishers. 2002. https://www.goodreads.com/work/best_book/863100-boys-in-white

36 Liberati EG, Tarrant C, Willars J, *et al.* How to be a very safe maternity unit: an ethnographic study. *Social Science & Medicine* 2019;**223**:64–72. doi:10.1016/j.socscimed.2019.01.035

37 LeCompte MD, Schensul JJ. *Designing & conducting ethnographic research: an introduction*. Lanham, MD: Rowman Altamira. 2010.

38 Darbyshire D, Brewster L, Isba R, *et al.* Retention of doctors in emergency medicine: a scoping review protocol. *JBI Evidence Synthesis* 2020;**18**:154–162.

39 Sanday PR. The ethnographic paradigm(s). *Administrative Science Quarterly* 1979;**24**:527–38. doi:10.2307/2392359

40 Atkinson P. *Thinking ethnographically*. London, UK: SAGE. 2017.

41 Grint K. *The sociology of work*. Fourth edition. Cambridge, UK: Polity Press. 2016.

42 Edgell S, Gottfried H, Granter E, *et al. The SAGE handbook of the sociology of work and employment*. 2016.

43 Hochschild AR. *The managed heart: commercialization of human feeling*. Berkerley, CA: University of California Press. 2012. http://ebookcentral.proquest.com/lib/lancaster/detail.action?docID=870020 (accessed 25 Jun 2019).

44 Woolcock M, Narayan D. Social capital: implications for development theory, research, and policy. *World Bank Res Obs* 2000;**15**:225–49. doi:10.1093/wbro/15.2.225

45 Slattery M. *Key ideas in sociology*. Oxford, UK: Nelson Thornes. 2003.

46 Wacquant L, Akçaoğlu A. Practice and symbolic power in Bourdieu: the view from Berkeley. *Journal of Classical Sociology* 2017;**17**:55–69. doi:10.1177/1468795X16682145

FUNDED BY NIHR National Institute for Health Research

For peer review only - http://bmjopen.bmj.com/site/about/guidelines.xhtml

47 Jafar AJN. What is positionality and should it be expressed in quantitative studies? *Emerg Med J* 2018;**35**:323–4. doi:10.1136/emermed-2017-207158

48 LeCompte MD, Goetz JP. Problems of reliability and validity in ethnographic research. *Rev Educ Res* 1982;**52**:31–60. doi:10.3102/00346543052001031

49 Rodgers BL, Cowles KV. The qualitative research audit trail: a complex collection of documentation. *Res Nurs Health* 1993;**16**:219–226.

50 Varpio L, Ajjawi R, Monrouxe LV, *et al.* Shedding the cobra effect: problematising thematic emergence, triangulation, saturation and member checking. *Med Educ* 2017;**51**:40–50. doi:10.1111/medu.13124

51 Given L, Saumure K. Trustworthiness. In: *The SAGE encyclopedia of qualitative research methods*. Thousand Oaks, CA: SAGE Publications. 2008. doi:10.4135/9781412963909.n470

52 O'Reilly K. *Key concepts in ethnography*. Thousand Oaks, CA: SAGE. 2008.

53 Pink S, Morgan J. Short-term ethnography: intense routes to knowing. *Symbolic Interaction* 2013;**36**:351–61. doi:10.1002/symb.66

54 Fetterman DM. *Ethnography: step by step*. 2nd ed. Thousand Oaks, CA: Sage Publications, Inc. 1998.

55 Kvale S, Brinkmann S. *InterViews*. 3rd ed. London, UK: SAGE Publications. 2014.

56 Silverman D. *Interpreting qualitative data*. 5th ed. London, UK: SAGE. 2015.

57 QRS. *nVivo 12 Mac*. QRS International. 2018.

58 NHS Data Dictionary. Accident and emergency department type. NHS Data Model and Dictionary Version 3. 2018.https://www.datadictionary.nhs.uk/data_dictionary/attributes/a/acc/accident_and_emergency_department_type_de.asp (accessed 5 Feb 2019).

59 Idelji-Tehrani S, Al-Jawad M. Exploring gendered leadership stereotypes in a shared leadership model in healthcare: a case study. *Medical Humanities* 2018;**45**:388–98. doi:10.1136/medhum-2018-011517

60 Brummell SP, Seymour J, Higginbottom G. Cardiopulmonary resuscitation decisions in the emergency department: An ethnography of tacit knowledge in practice. *Social Science & Medicine* 2016;**156**:47–54. doi:10.1016/j.socscimed.2016.03.022

61 Murphy E, Dingwall R. Informed consent, anticipatory regulation and ethnographic practice. *Social Science & Medicine* 2007;**65**:2223–34. doi:10.1016/j.socscimed.2007.08.008

62 Braun V, Clarke V. Reflecting on reflexive thematic analysis. *Qualitative Research in Sport, Exercise and Health* 2019;**11**:589–97. doi:10.1080/2159676X.2019.1628806

FUNDED BY NIHR National Institute for Health Research

For peer review only - http://bmjopen.bmj.com/site/about/guidelines.xhtml

63 Clarke V, Braun V. Thematic analysis. In: Lyons E, Coyle A, eds. *Analysing qualitative data in psychology*. London, UK: SAGE. 2016.

64 Braun V, Clarke V, Hayfield N, *et al.* Thematic analysis. In: Liamputtong P, ed. *Handbook of research methods in health social sciences*. Singapore: Springer Singapore 2019. 843–60. doi:10.1007/978-981-10-5251-4_103

65 Terry G. Doing Thematic Analysis. In: Lyons E, Coyle A, eds. *Analysing Qualitative Data in Psychology*. Thousand Oaks, CA: SAGE. 2016.

66 Rodgers BL. Audit trail. In: *The SAGE encyclopedia of qualitative research methods*. Thousand Oaks, CA: SAGE Publications, Inc. 2008. doi:10.4135/9781412963909.n25

67 Hobbs D. Ethnography. In: Jupp V, ed. *The SAGE Dictionary of Social Research Methods*. London, UK: SAGE Publications Ltd. 2006. https://methods.sagepub.com/reference/the-sage-dictionary-of-social-research-methods (accessed 18 Oct 2019).

68 Di Domenico M, Phillips N. Participant observation. In: *Encyclopedia of case study research*. Thousand Oaks, CA: SAGE Publications, Inc. 2010. doi:10.4135/9781412957397.n244

69 Rowe WE. Positionality. In: *The SAGE encyclopedia of action research*. Thousand Oaks, CA: SAGE Publications Ltd .2014. doi:10.4135/9781446294406.n277

70 May T, Perry B. *Reflexivity: the essential guide*. London, UK: SAGE. 2017.

71 Bloor M, Wood F. *Keywords in qualitative methods*. London, UK: SAGE Publications Ltd. 2006. doi:10.4135/9781849209403

Figure Captions

Figure 1. What is the James Lind Alliance? Information taken from the JLA website. http://www.jla.nihr.ac.uk/about-the-james-lind-alliance/ (accessed 15th January 2020)

Figure 2. Summary of the ethnography. The arrows suggest a linearity to the study which is not reflected in the reality of ethnographic practice, instead the image is aiming to convey the different workstreams that come together in the analysis. The figure also fails to include the messy connections between the different parts of the study and the overlap between the intended outputs.

Figure 3. The six stages of thematic analysis as applied to this study. An iterative process, it is expected that the analysis will go back and forth between stages a number of times.[63,66]

For peer review only

FUNDED BY NIHR National Institute for Health Research

The James Lind Alliance

The James Lind Alliance (JLA) is a non-profit making initiative established in 2004. It brings patients, carers and clinicians together in Priority Setting Partnerships (PSPs) to identify and prioritise the Top 10 unanswered questions or evidence uncertainties that they agree are the most important.

The aim of this is to make sure that health research funders are aware of the issues that matter most to the people who need to use the research in their everyday lives. It is part of the NIHR (National Institute for Health Research), the research funding arm of the United Kingdom's NHS
(National Health Service).

Figure 1
What is the James Lind Alliance? Information taken from the JLA website. http://www.jla.nihr.ac.uk/about-the-james-lind-alliance/ (accessed 15th January 2020)

1524x1016mm (96 x 96 DPI)

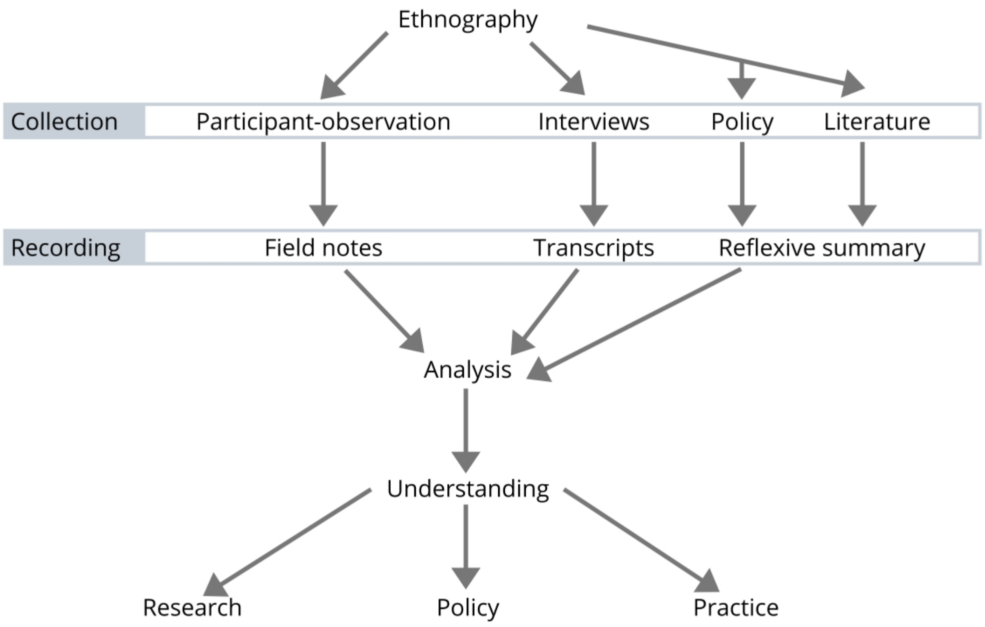

Figure 2. Summary of the ethnography. The arrows suggest a linearity to the study which is not reflected in the reality of ethnographic practice, instead the image is aiming to convey the different workstreams that come together in the analysis. The figure also fails to include the messy connections between the different parts of the study and the overlap between the intended outputs.

1524x1016mm (96 x 96 DPI)

Stage	Description	Applied to this study
1	**Familiarisation** – involves becoming immersed in the data, getting to know it. It is about noticing things that may (or may not) be important.	This will involve reading field notes, transcripts, and other documents, and making notes about connections, possibilities, and quirks.
2	**Generating codes** – attaching labels known as codes to portions of data to create meaning at the intersection between the data set and interpretative resource that is the researcher.	Using qualitative data analysis computer software (NVivo 12 for Mac) to attach codes to data. Coding choices will be documented and attached to each code.
3	**Generating initial themes** – a good theme both identifies the area of data and tells the researcher something about it. These themes will evolve or be discarded. Sometimes called candidate themes.	Stepping back form the fine detail of the data to try and group codes that inform a specific issue.
4	**Revising/reviewing themes** – involves reviewing themes against the coded data and the entire data set. Mismatches lead to revisions.	Compiling all data for each candidate theme and reviewing against the coded data and data set in turn.
5	**Defining and naming themes** – creatively attaching names to themes that signal the core of theme. This aims to move analysis to the conceptual level while maintaining links to the data.	Thinking about the audience and research objectively to name themes that can help effectively communicate the findings.
**6**	**Writing** – producing the output from the research. The writing tests the themes. How well do they work together and in isolation? How do they stand up to the literature? These challenges lead to returning to earlier analytical stages.	Writing up the doctoral thesis, papers for medical and sociological audiences, and producing summaries for policy makers and the public.

Figure 3. The six stages of thematic analysis as applied to this study. An iterative process, it is expected that the analysis will go back and forth between stages a number of times.[63,66]

For peer review only

Interview Guide

Where have all the doctors gone? An ethnographic study to explore the critical retention problem in Emergency Medicine

This guide will be used as a structure for interviews. It will allow the interviewer to draw out value from an interview and a structure to return to if the interview is going off topic. It will be shaped in response to specific observations in the fieldwork and tailored to the individual, with a focus on the overall research question: what makes doctors stay working in emergency medicine?

Pre-interview Checks

Ensure you have completed the consent procedure as outlined in the protocol.

Thank the participant for contributing.

Check the voice recording equipment is working.

Opening Question

To start the interview of ask some background questions, these will vary depending on your pre-existing knowledge of the participant.

- What is your role?
- How long have you worked in emergency medicine?
- What attracted you to work in emergency medicine?

This may direct the interview into retention, if not move onto directly asking about it.

What keeps you working in emergency medicine?

This is a very open question, follow up questions will depend upon the trajectory of the interview but may include:

Can you talk about a specific experience?
Prompts: what affected your decision? What happened after that experience?

Question Relating to Culture

How would you describe the culture of this emergency medicine department?
Is the culture here different to other EDs you have worked in?

For peer review only - http://bmjopen.bmj.com/site/about/guidelines.xhtml

Do you think that workplace culture affects staff retention?
How has it affected your work in emergency medicine?

Again, an open question, while generalities about retention and culture are interesting it is important as an interviewer to try and get to the personal experience.

Question Relating to Environment

How would you describe the work environment of this emergency medicine department?
Is the workplace here different to other EDs you have worked in?

How does the work environment affect your decision to work in EM?

Again, open questions, while generalities about retention and work environment are interesting it is important as an interviewer to try and get to the personal experience.

Digging for Depth

Having opened the conversation and grounded it in the interviewee's lived experience related to retention, the interviewer now needs to try and expand and develop any idea. Three tools are available for this:

1. *Being sensitive to cues from the previous questions and returning to them.*
2. *Asking about colleagues' experiences, building on specifics, and then returning to personal views about this.*
3. *Using a social media post to generate further discussion.*

This list is not exhaustive, nor it is a checklist. Some interviews may use all, some none, they are simply options that a skilled and attentive interviewer can apply to try and gain as much value from the interaction.

Post Interview Checklist

On completion of the interview a number of things need to be completed:

- Ensure the interviewee is alright, it is possible that the interview covered some difficult ground, the participant information sheet has sources of support.
- Thank participant for contributing.
- Are you ok? Remember the sources of support are available to the interviewer as well.
- Interviewees can be given a certificate of participation to include in their portfolio, taking part in research as a participant can help clinicians understand the process. This also acts as a formal thank you to participants.

Once the interviewee has vacated the interview space the interviewer should complete field notes related to the interview, the closer to the interview the better.

For peer review only - http://bmjopen.bmj.com/site/about/guidelines.xhtml

No.	Topic	Explanation and location
	**Title and abstract**	
S1	Title - Concise description of the nature and topic of the study Identifying the study as qualitative or indicating the approach or data collection methods	Title clearly states ethnographic approach, that this article is a study protocol and the problem of focus – retention of doctors in emergency medicine. Lines 4-5.
S2	Abstract - Summary of key elements of the study using the abstract format of the intended publication	Structured abstract appropriate to a protocol. Lines 22-48.
	**Introduction**	
S3	Problem formulation - Description and significance of the problem/phenomenon studied; review of relevant theory and empirical work; problem statement	Background and importance of problem outlined (lines 66-135), gap in the literature highlighted (lines 137-143).
S4	Purpose or research question - Purpose of the study and specific objectives or questions	Objectives and research questions clearly stated. Lines 156-163.
	**Methods**	
S5	Qualitative approach and research paradigm - Qualitative approach and guiding theory if appropriate; identifying the research paradigm is also recommended; rationale**	Ethnographic approach and possible theories outlined. Ethnographic paradigm highlighted. Lines 202-211.
S6	Researcher characteristics and reflexivity - Researchers' characteristics that may influence the research; potential or actual interaction between researchers' characteristics and the research	Highlighted as important in the protocol, will be detailed in the results. Lines 213-215.
S7	Context - Setting/site and salient contextual factors; rationale**	Site selection and rationale detailed. Lines 304-308.
S8	Sampling strategy - How and why research participants, documents, or events were selected; criteria for deciding when no further sampling was necessary (e.g., sampling saturation); rationale**	Sampling stratify for participant-observation and interview components detailed (lines 310-321). Discussion of theoretic saturation (lines 287-290).
S9	Ethical issues pertaining to human subjects - Documentation of approval by ethics review board and participant consent; other confidentiality and data security issues	Ethics approval documented (lines 417-419). Process of consent and capacity detailed (lines 323-382).
S10	Data collection methods - Types of data collected; details of data collection procedures including (as appropriate)	Plan to collect field notes (lines 262-263) and interview transcripts (lines 293-294) documented. Dates will be

For peer review only

	start and stop dates of data collection and analysis, iterative process, triangulation of sources/methods, and modification of procedures in response to evolving study findings; rationale**	included in report but are not stated in the protocol. Ethnographic studies are expected to evolve, how this occurs will be reported in the results but is not predicted in the protocol.
S11	Data collection instruments and technologies - Description of instruments and devices planned for data collection	Interview guides included as appendices. Use of voice recording software planned but not specified.
S12	Units of study - Number and relevant characteristics of participants, documents, or events included in the study; level of participation	Planned recruitment strategy outlined. Lines 284-286.
S13	Data processing - Methods for processing data prior to and during analysis, including transcription, data entry, data management and security, verification of data integrity, data coding, and anonymization/de-identification of excerpts	Recording and processing of field notes delineated. Interviews recorded and transcribed. Data stored and managed with nVivo 12 for Mac. Lines 293-295.
S14	Data analysis - Process by which inferences, themes, etc., were identified and developed, including the researchers involved in data analysis; usually references a specific paradigm or approach; rationale**	Data analysis plan, using reflexive thematic analysis, outlined. Lines 386-397.
S15	Techniques to enhance trustworthiness	Audit trail (lines 217-221), reflexivity and transferability discussed (lines 222-224).
	**Results/findings**	
S16	Synthesis and interpretation	Not predetermined in protocol.
S17	Links to empirical data	Not predetermined in protocol.
	**Discussions**	
S18	Integration with prior work, implications, transferability, and contribution(s) to the	Not predetermined in protocol.
S19	Limitations - Trustworthiness and limitations of findings	Anticipated limitations discussed. Lines 404-413.
	**Other**	
S20	Conflicts of interest - Potential sources of influence or perceived influence on study conduct and conclusions; how these were managed	Lines 459-461.
S21	Funding - Sources of funding and other support; role of funders in data collection, interpretation, and reporting	Lines 465-467.

For peer review only

Table 1. SRQR Table - Standards for Reporting Qualitative Research. The SRQR tool is designed for reporting of completed studies, as such certain aspects are merely acknowledged are requiring inclusion in the final report. The table allows the reader to map the study protocol to a recognised quality indicator. The topic descriptions have been maintained, but edited for brevity, the full description can be found in the original publication.[1]

For peer review only
